# Effects of Weight Loss and Moderate-Protein, High-Fiber Diet Consumption on the Fasted Serum Metabolome of Cats

**DOI:** 10.3390/metabo11050324

**Published:** 2021-05-18

**Authors:** Marissa R. Pallotto, Patrícia M. Oba, Maria R. C. de Godoy, Kirk L. Pappan, Preston R. Buff, Kelly S. Swanson

**Affiliations:** 1 168 Animal Sciences Laboratory, Division of Nutritional Sciences, University of Illinois, 1207 West Gregory Drive, Urbana, IL 61801, USA; marissa.pallotto@effem.com; 2 6 Animal Sciences Laboratory, Department of Animal Sciences, University of Illinois, 1207 West Gregory Drive, Urbana, IL 61801, USA; obapm@illinois.edu; 3 164 Animal Sciences Laboratory, Division of Nutritional Sciences and Department of Animal Sciences, University of Illinois, 1207 West Gregory Drive, Urbana, IL 61801, USA; mgodoy2@illinois.edu; 4 Metabolon, Morrisville, NC 27560, USA; kirk.pappan@owlstone.co.uk; 5 The Nutro Company, Franklin, TN 37067, USA; prestonbuff@gmail.com; 6 162 Animal Sciences Laboratory, Division of Nutritional Sciences, Department of Animal Sciences and Department of Veterinary Clinical Medicine, University of Illinois, 1207 West Gregory Drive, Urbana, IL 61801, USA

**Keywords:** feline metabolism, feline obesity, metabolomics

## Abstract

Feline obesity elicits a plethora of metabolic responses leading to comorbidities, with potential reversal during weight loss. The specific metabolic alterations and biomarkers of organ dysfunction are not entirely understood. Untargeted, high-throughput metabolomic technologies may allow the identification of biological components that change with weight status in cats, increasing our understanding of feline metabolism. The objective of this study was to utilize untargeted metabolomic techniques to identify biomarkers and gain mechanistic insight into the serum metabolite changes associated with reduced food intake and weight loss in overweight cats. During a four-wk baseline period, cats were fed to maintain body weight. For 18 wk following baseline, cats were fed to lose weight at a rate of ~1.5% body weight/wk. Blood serum metabolites were measured at wk 0, 1, 2, 4, 8, 12, and 16. A total of 535 named metabolites were identified, with up to 269 of them being altered (*p*- and q-values < 0.05) at any time point. A principal component analysis showed a continual shift in metabolite profile as weight loss progressed, with early changes being distinct from those over the long term. The majority of lipid metabolites decreased with weight loss; however, ketone bodies and small lipid particles increased with weight loss. The majority of carbohydrate metabolites decreased with weight loss. Protein metabolites had a variable result, with some increasing, but others decreasing with weight loss. Metabolic mediators of inflammation, oxidative stress, xenobiotics, and insulin resistance decreased with weight loss. In conclusion, global metabolomics identified biomarkers of reduced food intake and weight loss in cats, including decreased markers of inflammation and/or altered macronutrient metabolism.

## 1. Introduction

Pets are becoming more integral members of the family, with 68% of US households owning a pet and 38% of US households owning a cat in 2016 [1]. Unfortunately, there is an alarming incidence of obesity in companion animals in the US and obesity is now considered the most common nutritional disorder in pets [2]. A survey conducted by the Association for Pet Obesity Prevention reported that 58.9% of US cats, or 50.5 million, are overweight (28%) or obese (30.9%) [3]. To further complicate the issue, many owners have a skewed perception of what constitutes healthy pet weight [4,5]. A general classification defines an overweight cat as one weighing 10–20% over their ideal body weight (BW) and an obese cat weighing > 20% above their ideal BW [6]. Each unit increase of body condition score (BCS) above ideal (BCS = 5) is roughly 10–15% over ideal BW [7,8].

Similar to humans, feline obesity is associated with comorbidities that have detrimental effects on health. The traditional development of obesity is due to a positive imbalance between energy intake and energy expenditure [2]. Aspects of the domestication and humanization of pets also contribute to obesity. These risk factors include neutering [4,5,9], decreased physical activity, increased food intake, and access to highly palatable high-fat and/or high-carbohydrate diets [10,11,12]. While obesity prevention would ideally avoid these conditions, it is necessary to develop effective and safe obesity treatment methods. The recommendation for safe and reasonable weight loss for cats is 1% to 1.5% of BW lost per wk [13,14]. To safely avoid inducing hepatic lipidosis during weight loss, cats should eat at least 50% of their maintenance energy requirement (MER) [6]. Previous experimental and clinical trials have used caloric restrictions between 59% and 80% of MER without evidence of hepatic lipidosis [15].

Obesity is a complex and multifactorial disease not only involving genetics, but also environmental and lifestyle factors [16]. Metabolomics, similar to genomics and proteomics, may now be used to analyze samples in order to gain insight on global metabolite responses to stimuli and link phenotype with genotype [17]. Obesity is a disease that affects whole-body function and elicits a plethora of metabolic responses, yet the specific alterations in metabolism and organ dysfunction are not entirely understood [18]. Metabolomic assays may be used to identify biomarkers of disease and/or evaluate the effects of nutritional intervention [19]. Such assays may not only increase our understanding of host metabolism and physiology, but may lead to the development of metabolite panels for use in veterinary practice or by pet food professionals. In veterinary clinics, metabolite signature panels may serve to diagnose disease and aid in the lifestyle, nutritional, and pharmaceutical management of pets with obesity, type 2 diabetes mellitus (DM), and other metabolic abnormalities. Likewise, pet food researchers may use metabolomic tools and/or targeted metabolite panels to develop and assess therapeutic diets intended to improve metabolism, reduce clinical signs, and increase the quality of life of pets.

While the field of metabolomics has developed rapidly in regard to human health, much less is known about the global metabolite profiles of domestic cats [20,21,22]. Recent studies have evaluated nutritional interventions in cats and dogs, but few have described the metabolomic changes in those that are overweight or obese [23,24,25,26,27]. Given the lack of knowledge in the area in general, and the lack of studies and data in obligate carnivores, the objective of this study was to determine effects of weight loss and moderate-protein, high-fiber diet consumption on the fasted serum metabolome of cats. Although it would be impossible to predict many specific metabolites given the lack of research and knowledge in the area, we hypothesized that weight loss would beneficially alter the serum metabolite profile, including the reduction in markers of inflammation, immune response, and insulin resistance, and would alter lipid and protein metabolite profiles as well.

## 2. Results and Discussion

### 2.1. Food Intake, Weight Loss, Body Composition, and Global Serum Metabolomics

A complete description of the food intake, BW, body composition, serum chemistry, and fecal microbiota data of cats is available in our previous publication [28]. Briefly, food intake was significantly lower for wk 1–18 than during the baseline period. Food intake was not different from wk 8 to 18 (47.7 to 44.7 g/d [153.2 to 143.7 kcal ME/d]), but cats continued to lose weight. All cats lost weight and body fat as a result of caloric restriction. Mean BW (7.7 vs. 6.2 kg) and mean BCS (7.6 vs. 6.0) decreased significantly from wk 0 to wk 16. Mean fat mass was significantly lower at wk 8, 12, and 16 (2.4–1.8 kg) than at wk 0 (2.9 kg). Body fat percentage was also significantly lower at wk 8, 12, and 16 (36.8–30.7%) than at wk 0 (40.9%). Mean lean body mass was significantly lower at wk 12 and 16 (3.7 kg) than at wk 0 (3.9 kg). Mean bone mineral content was significantly lower at wk 12 and 16 (92.7 and 92.4 g, respectively) than at wk 0 (108.2 g). Most serum biochemical results remained within the respective reference ranges of the clinical laboratory throughout the study. The exception was the significantly higher creatinine concentrations (reference range, 0.4 to 1.6 mg/dL) from wk 1 (1.74 mg/dL) to 16 (1.91 mg/dL) than the concentration at wk 0 (1.59 mg/dL). Mean triglyceride concentrations were significantly lower at wk 1–16 than the concentration at wk 0 (56.0 mg/dL). Relative abundance of fecal Actinobacteria increased and Bacteroidetes decreased with weight loss. At the genus level, *Blautia*, *Dorea*, *Eubacterium*, *Oscillospira*, *Peptococcus*, and *Ruminococcus* increased with weight loss, while *Lactobacillus*, *Butyricicoccus*, and *Phascolarctobacterium* decreased. Alpha diversity (species richness) and beta diversity were not affected.

**Figure 1:**
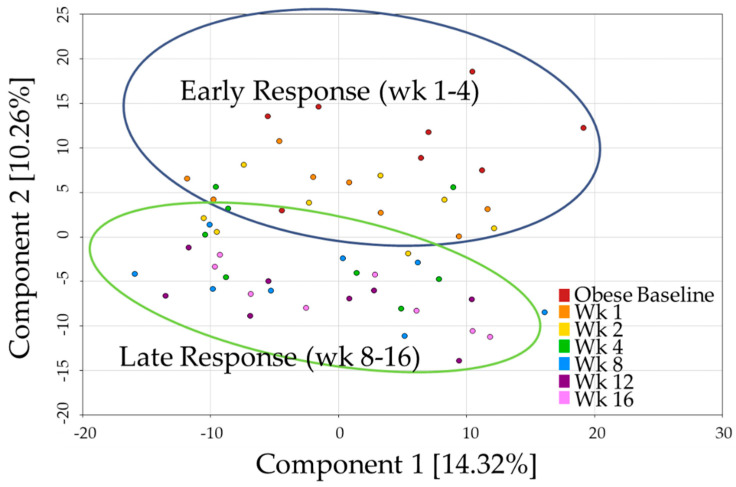
Principal component analysis of metabolite profiles demonstrates shifts that accompanied weight loss in cats.

A total of 535 named biochemicals were identified, with up to 269 metabolites being altered (*p*- and q-values < 0.05) at any time point. Principal component analysis (PCA, Figure 1) showed a continual shift in metabolite profile as weight loss progressed. Components one and two explained 14.3% and 10.3% of the variability, respectively. Although distinct clusters did not form, a biphasic relationship seemed to distinguish the early (wk 1–4) and late (wk 8–16) responses to weight loss. Broadly speaking, wk 1 and 2 appeared similar to baseline, wk 4 was variable, and wk 8, 12, and 16 were more differentiated from baseline. All of the metabolites discussed below were statistically significant with a combined *p*- and q-value ≤ 0.05.

**Figure 2:**
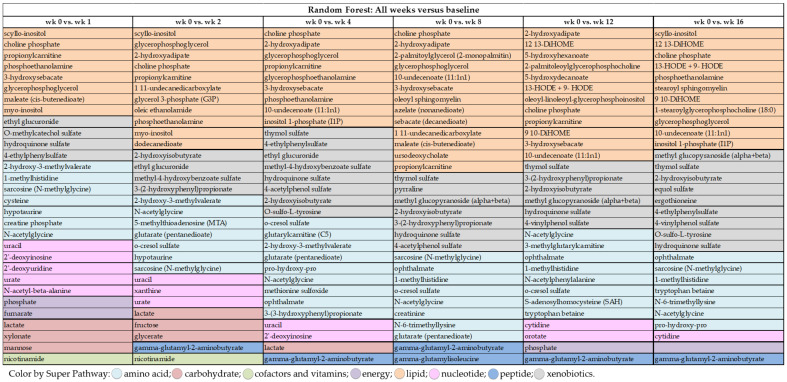
Top 30 serum metabolites differing between wk 1, 2, 4, 8, 12, and 16 of weight loss and baseline in cats as identified by random forest analysis.

**Figure 3:**
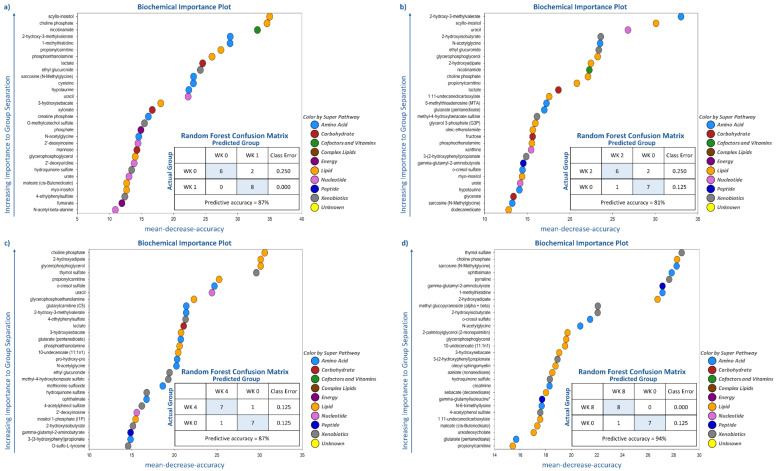

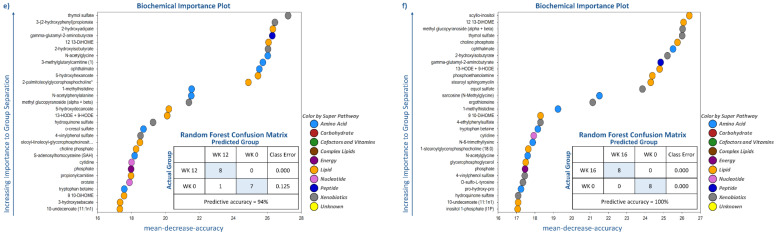
Top 30 serum metabolites that differed in cats and group prediction accuracy at (**a**) wk 1 versus baseline, (**b**) wk 2 versus baseline, (**c**) wk 4 versus baseline, (**d**) wk 8 versus baseline, (**e**) wk 12 versus baseline, and (**f**) wk 16 versus baseline, according to random forest analysis.

Random forest analysis was performed to identify and rank the top metabolites affected by weight loss by comparing metabolite profiles at wk 0 with the other time points (i.e., wk 1, 2, 4, 8, 12, and 16). As demonstrated by the large mean decrease accuracy (MDA) values in Figure 2 and Figure 3, Supplementary Tables S1–S4, weight loss quickly and dramatically altered metabolite profiles. Even though predictive accuracy improved with greater weight loss over time, accuracy was between 81% and 87% during the initial four wk. Random forest analysis showed that lipid- and amino acid-based metabolites made up 15 to 21 of the top 30 metabolites identified at each time point. Moreover, while some metabolites such as N-acetylglycine (amino acid (AA) metabolism), sarcosine (AA metabolism), choline phosphate (lipid metabolism), and 2-hydroxyisobutyrate (Figure 2 and Figure 3) had consistently high MDA values through the entire study, others were indicative of initial (e.g., uracil, lactate, nicotinamide, myo-inositol) or long-term (e.g., thymol sulfate; 1-methylhistidine; 12, 13-dihydroxyoctadecanoic acid (DiHOME); 9, 10-DiHOME) weight loss.

Many of the metabolites with the highest MDA in early weight loss (wk 1–4 vs. wk 0) were related to lipid metabolism (e.g., scyllo-inositol, choline phosphate, propionylcarnitine, phosphoethanolamine, Figure 2 and Figure 3). Other metabolites with consistently high MDA values were nicotinamide (cofactors and vitamins), 2-hydroxy-3-methylvalerate (AA metabolism), ethyl glucuronide (xenobiotic metabolism), lactate (carbohydrate metabolism), and uracil (nucleotide metabolism) (Figure 2 and Figure 3). Late weight loss (wk 8–16 vs. wk 0) was characterized by altering metabolites associated with lipid (10-undecenoate [11:1n1]; 12, 13-DiHOME; 9, 10-DiHOME), AA (ophthalmate and 1-methylhistidine), and xenobiotic (thymol sulfate and 2-hydroxyisobutyrate) metabolism (Figure 2 and Figure 3). One metabolite associated with peptide metabolism, gamma-glutamyl-2-aminobutyrate, had a consistently high MDA from wk 8–16 vs. wk 0 (Figure 2 and Figure 3).

### 2.2. Metabolite Profiles Associated with Lipid Metabolism

**Figure 4:**
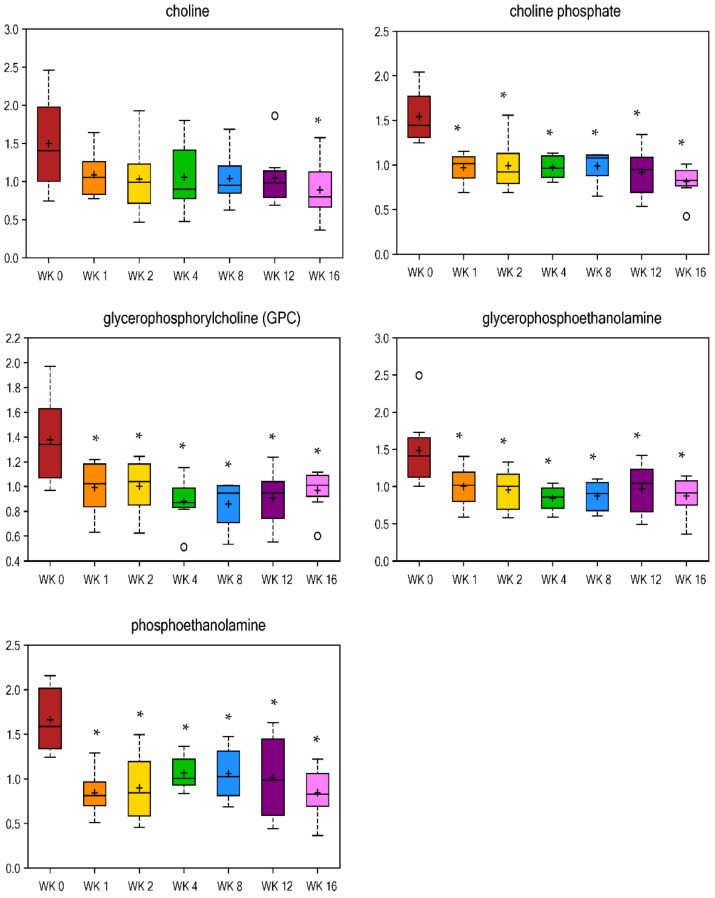
Fold change of metabolites related to phospholipids in cats during weight loss. * mean values were lower in comparison with wk 0 (*p* < 0.05).

Of the 269 metabolites altered with weight loss in this study, over half of them (i.e., 144) were related to lipid metabolism (Table 1 and Table 2, Supplementary Table S1). Metabolites of inositol (myo-inositol, scyllo-inositol, and inositol 1-phosphate, Table 2) and phospholipid metabolism (choline phosphate, phosphoethanolamine, and glycerophosphoethanolamine, Figure 4) immediately decreased by wk 1 of weight loss and remained lower at all wk vs. wk 0. In humans, abnormalities in myo-inositol metabolism have been associated with insulin resistance and its depletion has been found in tissues affected by diabetic microvascular and neurological complications in animal models and humans [29], but these metabolites have yet to be studied well in cats. Additionally, increases in myo-inositol may reduce insulin resistance [30,31,32,33,34]. When metabolized in the liver, fatty acids (FA) may be oxidized within the mitochondria to produce acetyl-CoA (generate ATP or sterols). FA may also be converted to triacylglycerols and exported as VLDL or stored as lipid droplets. Finally, FA may be metabolized into various phospholipids or sphingolipids [35]. The conversion of FA to sphingolipid or phospholipid metabolites has been linked to obesity, insulin resistance, type 2 DM, and cardiovascular disease [35,36,37]. Choline plays a role in the cell membrane structure, methyl metabolism, and lipid metabolism. The majority (>95%) of choline is used to synthesize phosphatidylcholine (PC) [38], which was shown to be greater in high-fat diet (HFD) fed obese mice [39]. Monoacylglycerols, mainly 2-palmitoylglycerol (wk 1 fold change = 3.05, Table 1), were increased by wk 1 of weight loss and remained higher throughout weight loss.

Long-chain FA and polyunsaturated FA (PUFA) were lower at wk 8, 12, and 16 vs. wk 0. The medium-chain FA 10-undeconoate (11: 1n1) was increased by wk 1 (fold change = 1.11) and remained higher at all wk vs. wk 0. Blood lipids may be derived from the diet, from adipose tissue, or the liver. These lipids are an important source of energy for the host and are stored primarily as triglycerides in adipose tissue [39]. Obesity is generally associated with elevated plasma, serum, and liver concentrations of non-esterified FA (NEFA), especially saturated FA (SFA) [40]. Furthermore, higher concentrations of stearic acid (*p* = 0.035), total SFA (*p* = 0.051), and palmitoleic acid (*p* = 0.068) along with lower linoleic acid (*p* = 0.084) concentrations have been reported in obese men [41]. Another study reported that total SFA (palmitic acid [C16:0] and stearic acid [C18:0]) and monounsaturated FA were lower (*p* < 0.005) after eight wk of energy restriction (–15% MER) in obese adults [42]. Although cats are true carnivores and do not develop cardiovascular disease in the same way that humans do, blood lipid profiles are indicative of their metabolic status and are important in regard to obesity and type 2 DM. Not surprisingly, most long-chain FA in this study were lower at wk 8, 12, and 16 vs. wk 0. Interestingly, monoacylglycerols, which are intermediates of lipolysis, were increased with weight loss.

Markers of primary bile acid metabolism, cholate, and taurocholate (Table 2), immediately and dramatically decreased with reduced food intake and weight loss, with a fold change of 0.03 and 0.14, respectively, at wk 1. Markers of secondary bile acid metabolism, deoxycholate, and ursodeoxycholate (Table 2), also decreased with weight loss. Bile acids do not only function to digest lipids in the diet, but are signaling molecules that regulate metabolism and inflammation in obesity, type 2 DM, dyslipidemia, and nonalcoholic fatty liver disease [43]. In humans, total bile acid concentrations are positively correlated with BMI [44] and with type 2 DM [45,46]. The same relationship may be expected in cats, but has not yet been studied to our knowledge. Mevalonate (Table 2), the product of rate-limiting enzyme HMG-CoA reductase (HMGR), was significantly lower after wk 8 vs. wk 0 (fold change = 0.65). Cholesterol, the major product of HMGR, remained unchanged. The cholesterol-derived primary and secondary bile acids were greatly decreased with weight loss. Cholate, in particular, was reduced by a fold change of 0.03 and 0.01 at wk 1 and wk 16 vs. wk 0, respectively. Taurocholate and deoxycholate had similar reductions. The bile acid precursor 7-Hoca was essentially unchanged, with an increase only at wk 8 vs. wk 0 (fold change = 1.1), suggesting a reduced need for emulsifying bile acids to aid in lipid digestion and absorption.

Markers of glycerolipid metabolism, glycerol 3-phosphate (G3P), and glycerophosphoglycerol (Table 2) decreased by wk 1 vs. wk 0 (fold change = 0.59 and 0.72, respectively). Some lysolipids decreased with weight loss, with many changes occurring at wk 4 or later. Many dicarboxylate FA (i.e., 2-hydroxyadipate and 1,11-undecanedicarboxylate, Table 2) decreased with weight loss. While markers of branched-chain amino acid (BCAA) metabolism (butyrylcarnitine and propionylcarnitine, Table 2) decreased, those of acylglycine and acylcarnitine metabolism increased throughout weight loss (Table 1). C3 acylcarnitine is a by-product of isoleucine and valine catabolism, while C5 acylcarnitines are intermediates of mitochondrial isoleucine and leucine catabolism. Both C3 and C5 acylcarnitines have been reported to increase with human obesity [47]. Furthermore, propionylcarnitine, butyrylcarnitine, and hexanoylcarnitines have been identified as being greater in obese men [41]. Butyrylcarnitine was lower from wk 2 to 16 vs. wk 0, and propionylcarnitine was lower at all wk vs. wk 0 in the current study.

Carnitine transports FA into the mitochondrion to produce energy via β-oxidation; therefore, carnitine is often used to promote weight loss [48]. Furthermore, obese mice and humans have displayed a depletion of carnitine in liver tissue [39,41]. It may be that lower carnitine in an obese state leads to the insufficient β-oxidation of NEFA, resulting in NEFA being stored as triglycerides in adipose tissue and ultimately, an accumulation of fat [17]. The majority of FA, including palmitate and stearate (Table 2), were decreased with weight loss. The ketone bodies acetoacetate and 3-hydroxybutyrate (BHBA) increased throughout the weight-loss period (Table 1). A study by Schmedes et al. reported an elevated (*p* < 0.001) concentration of the ketone bodies, BHBA, and acetoacetate, and a lower (*p* < 0.001) concentration of choline, glucose, tyrosine, and lactate in serum of overweight female subjects after a 6-wk very-low-calorie diet (average energy = 617 kcal/d) [49]. Carbohydrate restriction leads to a reduction in insulin secretion. When circulating insulin concentrations are low, stored fat in adipose tissue undergoes lipolysis [50,51]. Once hormone-sensitive lipase is liberated, NEFA in the hepatic mitochondria are preferentially used for β-oxidation to produce acetyl-CoA and ketone bodies, rather than the fats being esterified into triglycerides [52]. Decreased insulin release promotes a metabolic shift toward lipid oxidation and the utilization of FA and ketones for energy [53]. This is reflected by an increase in ketone bodies and a decrease in triglycerides in fasting serum samples. Our results in cats agree with these previous reports, with acetoacetate and BHBA increasing up to 1.93 and 2.09 fold, respectively (Table 1).

**Figure 5:**
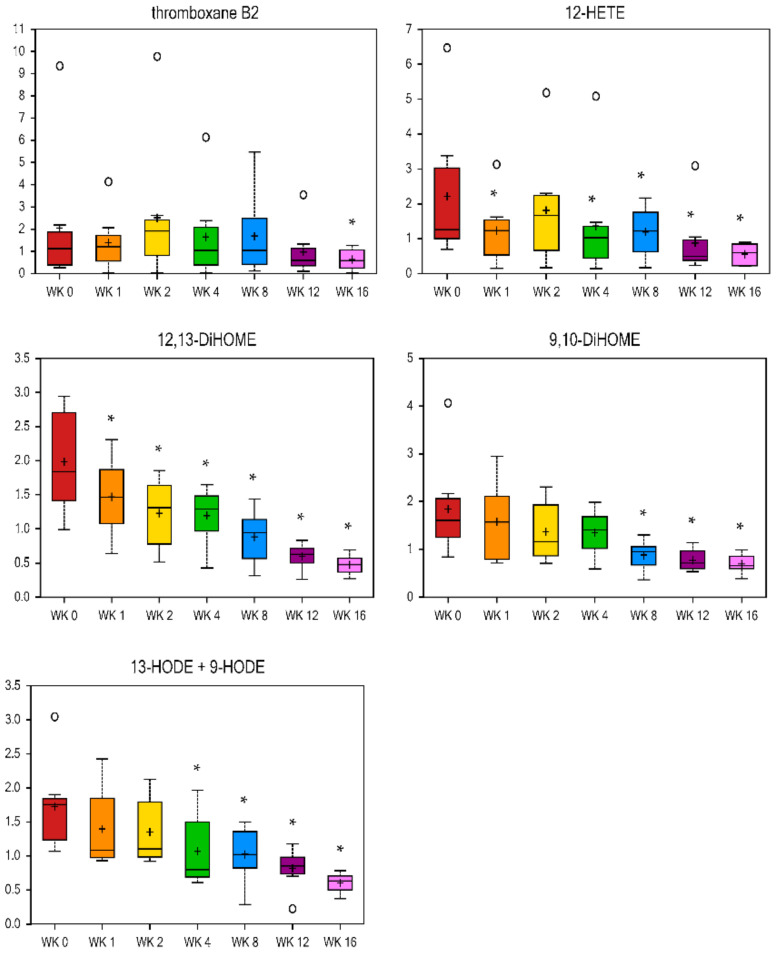
Fold change of metabolites related to eicosanoids and oxidized lipids in cats during weight loss. * mean values were lower in comparison to wk 0 (*p* < 0.05).

Eicosanoids are oxygenated bioactive metabolites derived from C-20 FA (including arachidonic acid), and include prostaglandins, thromboxanes, leukotrienes, and lipoxins [54]. They are mediators of acute inflammation, fever, and diseases such as cancer, atherosclerosis, and thrombosis. Therefore, preventing eicosanoid synthesis and action, or modifying the type of eicosanoid to be synthesized is the aim of many drugs. Eicosanoid-related metabolites were decreased with weight loss in the current study. Thromboxane B2 was lower at wk 16 vs. wk 0 (fold change = 0.31), and 12-hydroxyeicosatetraenoic acid (HETE) was reduced more quickly, with a fold change of 0.56 and 0.25 by wk 1 and wk 16 vs. wk 0, respectively (Figure 5). Triglyceride-rich lipoprotein (TGRL) lipolysis products cause inflammatory stimuli that possibly alter the endothelial barrier function and have pro-atherogenic and pro-inflammatory properties [55]. Monohydroxy FA (i.e., 3-hydroxysebacate, 5-hydroxyhexanoate, 5-hydroxydecanoate, and 13-HODE + 9-HODE, Figure 5, Table 2) and dihydroxy FA (12, 13-DiHOME and 9, 10-DiHOME, Figure 5) decreased with weight loss. Linoleic acid-derived 13-hydroxyl ocatadecadienoic acid (HODE) and 9-HODE are the major oxidized components of low-density lipoprotein (LDL) and very-low-density lipoprotein (VLDL), respectively [56,57]. Other products of linoleic acid oxidation include 12, 13-DiHOME, 9, 10-DiHOME, and epoxy octadecenoic acid (EpOME). Research by Wang et al. reported that significant amounts of these oxidized lipids are released during TGRL lipolysis [55]. During weight loss in the current study, 12, 13-DiHOME was primarily reduced, with a fold change of 0.74 and 0.24 at wk 1 and wk 16 vs. wk 0, respectively. The metabolite 9, 10-DiHOME was also lower at wk 8, 12, and 16 vs. wk 0, while 13-HODE and 9-HODE were lower from wk 4 to 16 vs. wk 0 of weight loss (Figure 5). These results in the study of cats agree with the human and rodent literature that obesity is a state of low-grade inflammation and that weight loss can reduce this state.

A correlation analysis identified 17 lipid metabolites from an untargeted analysis that were positively correlated with fasted serum triglyceride concentrations, and 4 lipid metabolites from an untargeted analysis were positively correlated with fasted serum cholesterol concentrations (Supplementary Table S5). Another 33 lipid metabolites, including ketone bodies (3-hydroxybutyrate and acetoacetate), were positively correlated with body fat mass and fat percentage. Finally, 26 lipid metabolites were positively correlated with BW (Supplementary Table S6).

### 2.3. Metabolite Profiles Associated with Amino Acid and Peptide Metabolism

Approximately 40% of the metabolites altered by reduced food intake and weight loss were related to AA metabolism (i.e., 100) and peptide metabolism (i.e., 14) (Table 3 and Table 4, Supplementary Table S2). The sub-pathways of AA metabolism had variable results, containing metabolites that both increased and decreased with weight loss. Most metabolites of lysine metabolism (i.e., N-6-trimethyllysine, glutarylcarnitine, and 3-methylglutarylcarnitine, Table 3) were higher at wk 1 or 2 vs. wk 0 and remained higher throughout the study. Conversely, glutarate was decreased by wk 1 vs. wk 0 (fold change = 0.75, Table 4) and remained lower at all wk vs. wk 0. Metabolites of glycine, serine, and threonine metabolism had differing results, with N-acetylglycine being higher at wk 1 vs. wk 0 (fold change = 1.37, Table 3), and sarcosine being lower (fold change = 0.62, Table 4). Glutamate was lower at wk 1, 2, and 16 versus wk 0 (fold change = 0.71, 0.73, and 0.67, respectively, Table 4), while glutamine was only higher at wk 12 vs. wk 0 (fold change = 1.19, Table 3). In obese children as compared to normal-weight children, glutamine was also increased with weight loss [58]. In humans, it was also reported that the association between obesity and the activation of the hexosamine pathway, which consumes glutamine upon the formation of glucosamine-6-phosphate from fructose 6-phosphate [59,60], and the glucosamine and hexosamines subsequently formed from it, are known to be associated with the development of insulin resistance [60,61]. There was an increase in 1-methylhistidine by wk 1 vs. wk 0 (fold change = 1.2, Table 3), and it remained higher at all wk vs. baseline. Most metabolites of phenylalanine and tyrosine metabolism were decreased with weight loss, with N-acetylphenylalanine being lower at wk 1 vs. wk 0 (fold change = 0.87), o-cresol sulfate being lower at wk 2 vs. wk 0 (fold change = 0.49), and others being lower at wk 4 vs. wk 0 (Table 4). Similarly, in humans, insulin resistance and an increased risk of developing type 2 DM are associated with elevated concentrations of aromatic AA (tyrosine and phenylalanine) and BCAA (isoleucine, leucine, and valine), with the aromatic AA and BCAA being reported to decrease after weight loss in obese individuals [47,62,63,64,65].

Metabolites of BCAA (leucine, isoleucine, and valine) metabolism were inconsistently changed by weight loss, with 2-hydroxy-3-methylvalerate being the only metabolite to decrease by wk 1 vs. wk 0 (fold change = 0.57, Table 4) and remain lower at all wk. Valine was not changed with weight loss in the current study, but leucine was lower at wk 12 vs. wk 0 (Table 4). High fasted BCAA and aromatic AA concentrations have been documented in obese humans [66] and are thought to contribute to obesity-related comorbidities such as insulin resistance and glucose intolerance [47]. Reportedly, obese men had plasma valine and leucine concentrations that were 23% and 14%, respectively, higher than lean men [41]. BCAA catabolism may be inhibited with obesity, as obese ob/ob mice and Zucker rats reportedly had depressed activities of BCAA aminotransferase and the branched-chain α-ketoacid dehydrogenase enzyme complex [67]. Conversely, HFD-fed mice have been shown to have lower serum BCAA concentrations [68,69]. In approximately 1300 humans aged 40–79, higher BCAA concentrations were related to older age, male sex, metabolic syndrome, obesity, cardiovascular disease risk, dyslipidemia, hypertension, and uric acid [70]. Medium- and long-chain acylcarnitines, by-products of the mitochondrial catabolism of BCAA, branched-chain keto acids, and BCAA distinguished obese people with insulin resistance from those without [71]. In a study of nearly 900 hypertension patients, BCAA, tyrosine, and phenylalanine were associated with metabolic syndrome and impaired fasting glucose [72]. Finally, elevations in BCAA and alanine were associated with insulin resistance, whereas higher concentrations of glutamine and glycine were associated with a lower likelihood of insulin resistance [73].

**Figure 6:**
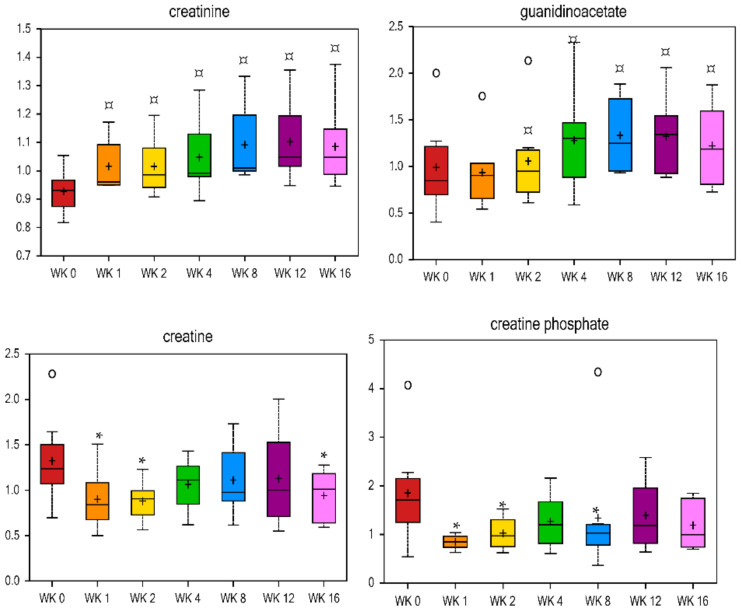
Fold change of metabolites related to creatinine metabolism in cats during weight loss. * mean values were lower in comparison with wk 0 (*p* < 0.05). ¤ mean values were higher in comparison with wk 0 (*p* < 0.05).

Metabolites of methionine, cysteine, S-adenosylmethionine (SAM), and taurine metabolism mostly decreased with weight loss, and methionine sulfoxide, S-adenosylhomocysteine (SAH), taurine, hypotaurine, and N-acetyltaurine all decreased by wk 1 vs. wk 0 (fold change = 0.73, 0.63, 0.72, 0.31, and 0.86, respectively, Table 4) and remained lower throughout weight loss. Methionine restriction in rodents reduced circulating lipids, increased metabolic flexibility, enhanced insulin sensitivity, and limited fat deposition by increasing the total daily energy expenditure [74,75,76,77,78,79,80]. Taurine plays a role in the conjugation of cholesterol and bile acids and has been thought to play a role in obesity [17]. In a previous study with Labrador Retriever dogs, a decrease in postprandial urinary taurine concentration indicated an alteration in lipid metabolism in overweight dogs, and that taurine may be a possible biomarker [27]. In humans, plasma total cysteine has a positive relationship with BMI, with higher total cysteine being present in overweight individuals [81,82,83,84,85]. Urea cycle metabolites such as urea and citrulline were lower at wk 8, 12, and 16 vs. wk 0 (Table 4), while pro-hydroxy-pro was higher at wk 2, 4, 8, 12, and 16 vs. wk 0 (Table 3). Markers of creatine metabolism had opposing results, with both creatine and creatine phosphate being lower at wk 1 and 2 vs. baseline, and creatinine and its precursor guanidinoacetate being higher at wk 1–16 and 4–16 vs. baseline, respectively (Figure 6).

Glutamine is the most abundant AA in plasma, and glycine is generated from serine, which is derived from pyruvate. Both of these AA, which are precursors of urea biosynthesis and glucose metabolism [86], were previously reported to be lower (*p* < 0.05) in obese individuals [87,88]. Protein restriction has been reported to increase glycine and serine concentrations [89]. Serine, glycine, and threonine metabolic pathways were significantly higher in humans that had Roux-en-Y gastric bypass surgery and sustained weight loss (RYGB-SWL) compared to ones who regained BW (RYGB-WR); most of the statistically different metabolites between the RYGB-SWL and RYGB-WR groups were involved in AA metabolism, one-carbon metabolism, and nucleotide metabolism [90]. Similarly, in the current study, metabolites of glycine, serine, and threonine were observed to be higher at all wk during weight loss vs. wk 0.

Arginine and glycine synthesize creatine, which is broken down in skeletal muscle to produce creatinine [91]. Previous research has shown an increase of creatinine in the urine of obese humans (*p* < 0.01) [92] and in the serum of HFD-fed mice (*p* < 0.05) [69]. Ophthalmate, a metabolite of glutathione metabolism, was doubled at wk 4 (fold change = 2.02) in the current study and remained higher with weight loss (Table 3). In general, most peptide-related metabolites were increased with weight loss. Particularly noteworthy are gamma-glutamylisoleucine, gamma-glutamyl-2-aminobutyrate, N-acetylcarnosine, and prolylglycine, which were higher at wk 1 vs. wk 0 (fold change = 1.23, 1.41, 1.24, and 1.27 respectively) and remained higher at all wk (Table 3).

A correlation analysis identified five amino acid and one peptide metabolites from an untargeted analysis that were negatively correlated with fasted serum cholesterol concentrations; two amino acid and one peptide metabolites from an untargeted analysis that were negatively correlated with fasted serum triglyceride concentrations; nine amino acid and two peptide metabolites from an untargeted analysis that were positively correlated with fasted serum creatinine concentrations; and eleven amino acid and one peptide metabolites from an untargeted analysis that were positively correlated with fasted serum blood urea nitrogen concentrations (Supplementary Table S5). Additionally, ten amino acid and one peptide metabolites were negatively correlated with body fat percentage, while six amino acid and one peptide metabolites were negatively correlated with BW (Supplementary Table S6). The gut microbiome can influence the blood metabolites of the host, including indole-containing metabolites, phenyl derivatives, and flavones [93]. It was previously reported that enteric bacteria (*Escherichia coli*, *Clostridium sporogenes*) can convert tryptophan to indoles, and *Bifidobacterium infantis* can increase plasma levels of tryptophan [94,95,96]. In the present study, body fat percentage was negatively correlated with two tryptophan-based metabolites, and positively correlated with tryptophan and one phenylalanine/tyrosine-based metabolite (Supplementary Table S6). These results may be due to substrate availability and/or the alteration of gut bacteria. However, further studies are needed to evaluate the effects of dietary composition, food intake, and weight loss on the relationships among host metabolites and the gut microbiota. An improved understanding may consequently allow for improved dietary interventions for the treatment of obesity in cats.

### 2.4. Metabolite Profiles Associated with Carbohydrate and Energy Metabolism

**Figure 7:**
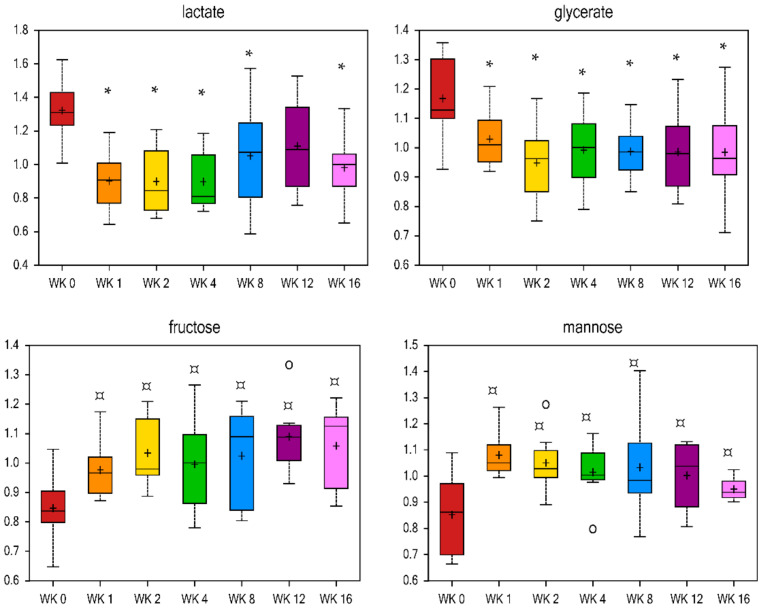
Fold change of metabolites related to carbohydrates in cats during weight loss. * mean values were lower in comparison with wk 0 (*p* < 0.05). ¤ mean values were higher in comparison with wk 0 (*p* < 0.05).

Reduced food intake and weight loss affected nine and seven metabolites related to carbohydrate and energy metabolism, respectively (Table 5, Supplementary Table S3). Lactate and glycerate were decreased by wk 1 (fold change = 0.68 and 0.88, respectively) and remained lower with weight loss, while fructose and mannose were increased by wk 1 (fold change = 1.15 and 1.27, respectively) and remained higher with weight loss (Figure 7). Previous research has demonstrated that subcutaneous fat is a source of lactate [97] and that HFD-induced obese mice or obese Zucker rats lacking the leptin receptor have a higher concentration of lactate in the urine, blood, and liver tissue [69,98,99,100,101]. Because lactate is a precursor of gluconeogenesis, greater plasma lactate in obese models may reflect alterations in hepatic glucose and lipid metabolism. The decrease in serum lactate and glycerate observed in the current study may indicate a beneficial shift in glucose metabolism with weight loss. Fructose is converted to glycerol and acyl groups for synthesis of triglycerides in the liver [102]. Higher fructose at all wk vs. wk 0 agrees with the previously discussed decreases in fasting serum triglyceride concentrations [28]. Citrate was only higher at wk 16 vs. wk 0 (fold change = 1.09, Table 5), and alpha-ketoglutarate was only lower at wk 2 vs. wk 0 (fold change = 0.81, Table 5). Fumarate and phosphate were lower at all wk vs. wk 0 (Table 5). The literature suggests that alpha-ketoglutarate is a positive predictor of obesity [103], yet alpha-ketoglutarate was only lower at wk 2 vs. wk 0 in the current study. Pyruvate enters the tricarboxylic acid cycle (TCA) via citrate, which is regulated in the plasma by insulin, glucose, FA utilization, cholesterol synthesis, and liver clearance and excretion [68]. Plasma citrate has been reported to be higher in diabetic rats [104] and HFD-fed obese mice [68], and lower with insulin administration in children [105]. In contrast, lower serum citrate has been reported in humans with type-2 DM [106].

A correlation analysis identified one carbohydrate and one energy metabolites from an untargeted analysis that were positively correlated with fasted serum cholesterol concentrations, while two carbohydrate metabolites from an untargeted analysis were positively correlated with fasted serum triglyceride concentrations (Supplementary Table S5). Another energy metabolite was negatively correlated with body fat percentage (Supplementary Table S6).

### 2.5. Metabolite Profiles Associated with Nucleotide, Xenobiotic, and Cofactor and Vitamin Metabolism

**Figure 8:**
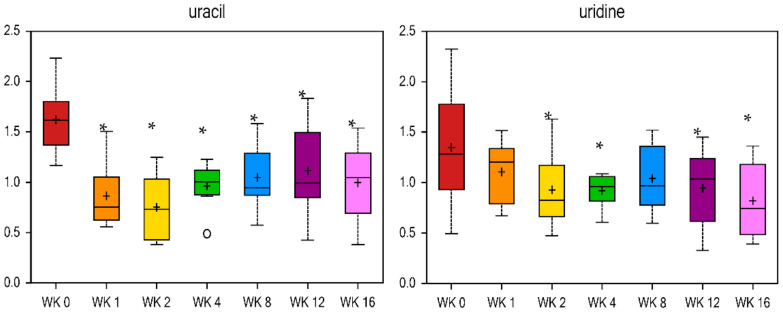
Fold change of metabolites related to pyrimidine metabolism in cats during weight loss. * mean values were lower in comparison with wk 0 (*p* < 0.05).

The remaining metabolites altered by reduced food intake and weight loss were related to nucleotide (26 metabolites), xenobiotic (38 metabolites), and cofactor and vitamin (19 metabolites) metabolism (Table 6 and Table 7, Supplementary Table S4). Markers of xanthine- or inosine-containing purine metabolism were decreased, with xanthine, 2′-deoxyinosine, and urate quickly decreasing by wk 1 vs. wk 0 (fold change = 0.48, 0.46, and 0.74, respectively, Table 7). Some markers of pyrimidine metabolism such as uracil (Figure 8), 2′-deoxyuridine, and cytidine were decreased (Table 7), while others such as orotate were increased (Table 6) with weight loss. It has been shown that uridine infusion induced insulin resistance in rats [107] and has been correlated with insulin resistance in hypertensive patients [108]. Results of the current study show lower uridine at wk 2, 4, 12, and 16 vs. wk 0 (fold change = 0.69, 0.68, 0.7, and 0.61, respectively). Uracil forms uridine when it is combined with a sugar ribose by a glycosidic linkage [109]. Uracil was lower at all wk vs. wk 0, with a fold change of 0.53 at wk 1 vs. wk 0 (Figure 8). Most xenobiotics decreased with weight loss. Markers of benzoate metabolism, such as 4-ethylphenylsulfate and 4-vinylphenol sulfate, were lower at wk 1 vs. wk 0 (fold change = 0.41 and 0.49) and continued to decrease with weight loss (Table 7). Similar results were observed in xenobiotics related to food and plant components (i.e., ergothioneine and pyrraline), drugs (i.e., 4-acetylphenol sulfate and hydroquinone sulfate), and chemicals (i.e., O-sulfo-l-tyrosine and ethyl glucuronide), with all being decreased with weight loss (Table 7). Conversely, the chemical-related xenobiotic 2-hydroxyisobutyrate increased with weight loss (fold change = 1.87 at wk 16 vs. wk 0, Table 6).

Correlation analysis identified five xenobiotic metabolites from an untargeted analysis that were negatively correlated with fasted serum cholesterol concentrations, two xenobiotic and one nucleotide metabolites from an untargeted analysis that were positively correlated with fasted serum triglyceride concentrations, four xenobiotic and one nucleotide metabolites from an untargeted analysis that were positively correlated with fasted serum creatinine concentrations, and fifteen xenobiotic and two nucleotide metabolites from an untargeted analysis that were positively correlated with fasted serum blood urea nitrogen concentrations (Supplementary Table S5). Likewise, three xenobiotic and one nucleotide metabolites were negatively correlated with body fat percentage, and six xenobiotic and one nucleotide metabolites were negatively correlated with BW (Supplementary Table S6).

In summary, this study used untargeted metabolomic analyses to identify hundreds of serum metabolites affected by weight loss in cats. Most of the metabolites identified from this study have not been reported previously. Because the cat is a strict carnivore, its metabolism is quite different from that of humans and rodent models. Therefore, these data are expected to serve as a foundation for future studies focused on feline obesity, metabolism, and health. A random forest analysis comparing metabolite profiles across time points demonstrated that lipid- and amino acid-based metabolites made up one-half to two-thirds of the top 30 metabolites most influential in regard to predictive accuracy. Our analyses also show that while some metabolites such as N-acetylglycine (amino acid metabolism), sarcosine (amino acid metabolism), and choline phosphate (lipid metabolism) were highly predictive of weight loss throughout the entire study, other metabolites were indicative of initial (e.g., uracil; lactate; nicotinamide; myo-inositol) or long-term (thymol sulfate; 1-methylhistidine; 12, 13-DiHOME; 9, 10-DiHOME) weight loss. The majority of metabolites associated with lipid metabolism decreased with weight loss, which was likely due to a reduction in food intake (diet acid-hydrolyzed fat = 8.9%, and caloric restriction 33–40% of baseline intake, wk 5–10, respectively). However, ketone bodies and small lipid particles (monoacylglycerol, FA, and medium-chain FA) were increased, indicating that obese cats undergoing weight loss use lipolysis and FA oxidation to produce energy. The majority of metabolites associated with carbohydrate metabolism were decreased with weight loss, which was thought to be due to lower intake. Metabolites associated with protein metabolism had a variable result with weight loss, which may indicate that cats are in a state of constant flux in regard to muscle mass loss and synthesis during energy deficit. Finally, metabolic mediators of inflammation, oxidative stress markers, xenobiotics, and biomarkers of insulin resistance were decreased with weight loss. This dataset provides an improved understanding of feline metabolism, with a specific focus on how it is impacted by reduced food intake and weight loss. Not only did this study identify biomarkers of weight loss in general, but many that are indicative of the early or late phases of weight loss, which may serve as a foundation for future research using targeted analysis. Such research may lead to the development of metabolite signature panels with an application in veterinary practice, whereby biomarkers may help diagnose disease and aid in the lifestyle, nutritional, and pharmaceutical management of obese cats. Targeted metabolite panels may also be used to develop and test therapeutic diets intended to improve metabolism and reduce clinical signs of obese cats.

## 3. Materials and Methods

### 3.1. Animals and Diet

Eight neutered adult male domestic shorthair cats with a mean BW = 7.7 ± 0.42 kg and mean BCS = 7.6 ± 0.38 on a 9-point scale [110] were used. Mean age at the start of the study was 7.78 ± 0.03 years. Cats were housed in a particular temperature (20 °C) and light-controlled (16 h light: 8 h dark cycle) room at the University of Illinois. Cats were individually housed for two 2 h periods each day during feedings to allow for individual food intake records. During the other 20 h/d, cats were group housed and allowed to socialize with one another and exercise outside of their cages in the room. All animal procedures were approved by the University of Illinois Animal Care and Use Committee prior to animal experimentation. All cats were fed a dry commercial diet (Nutro Veterinary Nutrition Feline Weight Loss Adult Chicken & Whole Brown Rice Formula, The Nutro Company, Franklin, TN, USA) throughout the duration of the study, which was formulated to meet nutrient recommendations for adult domestic cats in accordance with the National Research Council [111]. Water was available ad libitum at all times.

This experiment was performed as a repeated-measures design. The first 4 wk of the study represented the baseline period, when all cats were fed to maintain their starting BW. After baseline (wk 0), BW was measured twice a wk and food intake was adjusted to target weight loss at approximately 1.5% BW/wk. Because this colony of cats were previously fed to maintain a healthy BW and BCS, an appropriate estimation of the ideal BW and MER to maintain BW was known. Cats were fed individually twice a day from 8:30 a.m. to 10:30 a.m., and from 3:00 p.m. to 5:00 p.m. in their assigned cages. Any uneaten food was weighed and recorded at the end of the feeding period. Daily food intake, twice-weekly BW, and weekly BCS were recorded throughout the study.

### 3.2. Blood Collection and Serum Preparation

Overnight fasted (at least 12 h) blood samples (5 mL) were collected via radial, femoral, or jugular venipuncture at wk 0, 1, 2, 4, 8, 12, and 16. Animals were restrained, but sedation was not necessary because procedures were familiar to the cats and stress was minimal. Blood was collected into evacuated tubes (BD Vacutainer serum separator tubes, Becton, Dickinson, and Company, Franklin Lakes, NJ, USA) and allowed to clot at room temperature. All tubes were centrifuged at 13,000× *g* for 15 min at 4 °C. The supernatant (serum) then was pipetted into cryogenic vials. Samples were stored at −80 °C until further analysis.

Serum was analyzed by Metabolon (Metabolon, Inc., Durham, NC, USA) to evaluate changes in global metabolite profiles and to identify markers of weight loss. Samples were shipped on dry ice and immediately stored at −80 °C upon arrival. Each sample was inventoried into the Metabolon Laboratory Information Management System (LIMS) and assigned a unique identifier to track all handling, tasks, and results. Samples were prepared using the automated MicroLab STAR^®^ system (Hamilton Company, Salt Lake City, UT, USA). For quality control purposes, a recovery standard was added prior to the first step of the extraction process. To remove protein, dissociate small molecules bound to protein or trapped in the precipitated protein matrix, and to recover chemically diverse metabolites, proteins were precipitated with methanol under vigorous shaking for 2 min (GenoGrinder 2000, Glen Mills, Clifton, NJ, USA) followed by centrifugation. The resulting extract was divided into fractions for analysis by liquid chromatography–mass spectroscopy (LC–MS) and gas chromatography–mass spectroscopy (GC–MS), and a fraction was reserved for backup. Samples were placed briefly on a TurboVap^®^ (Thermo Fisher Scientific Inc., Waltham, MA, USA) to remove the organic solvent. For LC, the samples were stored overnight under nitrogen before preparation for analysis. For GC, each sample was dried under vacuum overnight before preparation for analysis.

### 3.3. GC/MS and LC/MS/MS Analysis

Ultrahigh Performance Liquid Chromatography–Tandem Mass Spectroscopy (UPLC–MS/MS): The LC/MS portion of the platform was based on a Waters ACQUITY ultra-performance liquid chromatography (UPLC) and a high resolution/accurate mass spectrometer (Thermo Fisher Scientific Inc., Waltham, MA, USA). The sample extract was dried and then reconstituted, in acidic or basic LC-compatible solvents. One aliquot was analyzed using acidic positive ion optimized conditions and the other using basic negative ion optimized conditions, in two independent injections using separate dedicated columns (Waters UPLC BEH C18-2.1 × 100 mm, 1.7 µm). Extracts reconstituted in acidic conditions were gradient-eluted from a C18 column using water and methanol containing 0.1% formic acid. The basic extracts were similarly eluted from C18 using methanol and water but with 6.5 mM ammonium bicarbonate. The third aliquot was analyzed via negative ionization, following elution from a HILIC column (Waters UPLC BEH Amide 2.1 × 150 mm, 1.7 µm) and using a gradient consisting of water and acetonitrile with 10 mM ammonium formate. The MS analysis alternated between MS and data-dependent MS/MS scans using dynamic exclusion (scan range = 80–1000 *m*/*z*).

The samples destined for analyses by GC–MS were dried under vacuum for a minimum of 18 h prior to being derivatized under dried nitrogen using bistrimethyl-silyltrifluoroacetamide (BSTFA). Derivatized samples were separated on a 5% diphenyl/95% dimethyl polysiloxane fused silica column (20 m × 0.18 mm ID; 0.18 um film thickness) with helium as a carrier gas and a temperature ramp from 60 °C to 340 °C in a 17.5 min period. Samples were analyzed on a Thermo Finnigan Trace DSQ fast-scanning single quadrupole mass spectrometer using electron impact ionization (EI) and operated at unit mass resolving power (scan range = 50–750 *m*/*z*).

Three types of controls were analyzed in concert with the experimental samples: a pooled matrix sample generated from a small volume of each sample served as a technical replicate throughout the data set; extracted water samples served as process blanks; and a cocktail of quality control (QC) standards spiked into every analyzed sample for instrument performance monitoring and chromatographic alignment. Instrument variability was determined by calculating the median relative standard deviation (RSD) for the standards that were added to each sample, prior to injection into the mass spectrometers. The RSD for this study was 4%. Overall process variability for this study was 11% and was determined by calculating the median RSD for all endogenous metabolites (i.e., non-instrument standards) present in 100% of the pooled matrix samples. Experimental samples were randomized across the platform run with QC samples spaced evenly among the injections.

Raw data were extracted, peak-identified, and QC processed with Metabolon’s hardware and software. Metabolites were identified by comparison to library entries of purified standards or recurrent unknown entities. Identification of known chemical entities was performed by comparing to Metabolon’s reference library entries of purified standards. Biochemical identifications were based on three criteria: retention index (RI) within a narrow RI window of the proposed identification, accurate mass match to the library +/− 0.005 amu, and the Mass Spectral (MS) MS/MS forward and reverse scores between the experimental data and authentic standards. The MS/MS scores were based on a comparison of the ions present in the experimental spectrum to the ions present in the library spectrum. Currently, more than 3300 commercially available purified standard compounds have been acquired and registered into Metabolon Sunquest Mitogen™ LIMS (Sunquest Information Systems, Tucson, AZ, USA) for distribution to both the liquid chromatography (LC) and gas chromatography (GC) platforms for the determination of their analytical characteristics. Additional mass spectral entries have been created for structurally unnamed biochemicals, which were identified by virtue of their recurrent nature (both chromatographic and mass spectral).

Curation procedures using Metabolon proprietary visualization and interpretation software were carried out to ensure that a high quality data set was made available for statistical analysis and data interpretation. The QC and curation processes were designed to ensure accurate and consistent identification of true chemical entities, and to remove those representing system artifacts, mis-assignments, and background noise. Library matches for each compound were checked for each sample and corrected if necessary. Peaks were quantified using area-under-the-curve. For studies spanning multiple days, a data normalization step was performed to correct variation resulting from instrument inter-day tuning differences. Each compound was corrected in run-day blocks by registering the medians to equal one (1.00) and normalizing each data point proportionately (termed the “block correction”). For studies that did not require more than one day of analysis, no normalization was necessary other than for purposes of data visualization.

### 3.4. Statistical Analysis

All data were analyzed by use of statistical software [112]. The experimental design consisted of a single factor (wk) experiment with repeated measures, with cat and wk as fixed effects. Differences among treatments were determined using a Fisher-protected least significant difference (LSD) with a Tukey adjustment to control for experiment-wise error. A probability of *p* ≤ 0.05 was accepted as statistically significant.

To evaluate changes in global metabolic profiles due to weight loss, a heat map was made using 535 normalized known metabolites. Hierarchical clustering was used to show large-scale differences in metabolic patterns, and the determination of distinct clusters was done using Array Studio with complete linkage and distance correlation settings. PCA was performed using all named metabolites to provide a simultaneous comparison of metabolic alterations that accompanied weight loss. Random forest (RF) analyses were performed to provide an estimate of how well individuals may be classified in the dataset. For a given decision tree, a random subset of data was selected to build a tree (“bootstrap sample”), and the remaining data, the “out-of-bag” (OOB) variables, were passed through the tree to obtain a class prediction for each sample. After the process was repeated thousands of times, a forest was produced. The final classification of each sample was determined by computing the class prediction frequency for the OOB variables over the whole forest; therefore, the OOB error rate is a measure of prediction accuracy. A total of 21 comparisons were made over time (0, 1, 2, 4, 8, 12, or 16 wk), with two groups being compared at a time. To determine which variables (metabolites) made the largest contribution to the classification, the MDA was determined by randomly permuting a variable, running the observed values through the trees, and then reassessing the prediction accuracy. If a variable was important to the classification, the prediction accuracy dropped after such a permutation. Thus, the RF analysis provided an importance rank ordering of metabolites. The top 30 metabolites were reported for each comparison.

A one-way analysis of variance (ANOVA) with repeated measures identified metabolites that changed with weight loss. An estimate of a false discovery rate (q-value) was calculated to take into account multiple comparisons. A combination of *p*- and q-value ≤ 0.05 was used to declare statistical significance. Statistical analyses were performed using the program “R” (http://cran.r-project.org/, accessed on 5 February 2015) and JMP (SAS Inst. Inc., Cary, NC, USA: http://www.jmp.com, accessed on 5 February 2015). Metabolite–physiologic data correlations were calculated using Pearson correlation coefficients. Data were reported as means with *p* < 0.05 for DEXA scan results and metabolite, and *p* < 0.0001 for fasted blood serum chemistry and metabolites were considered significant.

## Figures and Tables

**Table 1:** Serum metabolites related to lipid metabolism that were increased in cats undergoing weight loss.

Metabolic Pathway	Metabolite	Fold Change					
		wk 1	wk 2	wk 4	wk 8	wk 12	wk 16
		wk 0	wk 0	wk 0	wk 0	wk 0	wk 0
Medium Chain Fatty Acid	10-undecenoate (11:1n1)	1.11	1.21	1.40	1.52	1.48	1.60
Fatty Acid Synthesis	malonylcarnitine	1.21	1.35	1.57	1.77	1.84	1.58
	malonate (propanedioate)	1.36	1.44	1.38	1.30	1.37	1.36
	2-methylmalonyl carnitine	1.22	1.40	1.68	1.96	2.14	1.86
Fatty Acid Metabolism (Acyl Glycine)	hexanoylglycine	1.55	1.56	1.98	2.22	2.33	2.11
	N-octanoylglycine	1.22	1.3	1.55	3.04	2.78	1.70
Fatty Acid Metabolism (Acyl Carnitine)	Hydroxybutyrylcarnitine	0.94	1.13	1.32	1.85	2.00	1.63
Ketone Bodies	acetoacetate	1.54	1.59	1.93	1.91	1.54	1.08
	3-hydroxybutyrate (BHBA)	1.51	1.72	1.95	2.09	2.00	1.74
Monoacylglycerol	1-palmitoylglycerol (1-monopalmitin)	1.41	1.5	1.69	1.85	1.68	1.74
	2-palmitoylglycerol (2-monopalmitin)	3.05	1.81	2.18	2.74	2.59	1.92
	1-linoleoylglycerol (1-monolinolein)	1.37	1.65	1.81	1.59	1.45	1.65
	1-arachidonylglycerol	1.79	2.35	2.73	2.45	2.18	2.41
	1-docosahexaenoylglycerol	1.52	2.31	2.53	2.15	1.76	2.26
Sphingolipid Metabolism	stearoyl sphingomyelin	1.07	1.09	1.19	1.35	1.24	1.37
	oleoyl sphingomyelin	1.11	1.13	1.33	1.32	1.24	1.35

Numbers in red text were significantly higher than the baseline (wk 0).

**Table 2:** Serum metabolites related to lipid metabolism that were decreased in cats undergoing weight loss.

Metabolic Pathway	Metabolite	Fold Change					
		wk 1	wk 2	wk 4	wk 8	wk 12	wk 16
		wk 0	wk 0	wk 0	wk 0	wk 0	wk 0
Long Chain Fatty Acid	pentadecanoate (15:0)	0.93	0.90	0.85	0.80	0.80	0.81
	palmitate (16:0)	1.00	0.99	0.96	0.87	0.87	0.90
	margarate (17:0)	0.97	0.98	0.88	0.76	0.81	0.77
	stearate (18:0)	1.02	1.03	0.96	0.85	0.88	0.87
	arachidate (20:0)	1.04	1.06	0.97	0.82	0.86	0.86
Polyunsaturated Fatty Acid (n3 and n6)	stearidonate (18:4n3)	0.91	0.87	0.81	0.66	0.55	0.49
	eicosapentaenoate (EPA; 20:5n3)	0.95	0.94	0.86	0.70	0.65	0.74
	linoleate (18:2n6)	0.96	0.96	0.88	0.83	0.82	0.85
	linolenate (18:3n3 or 6)	0.88	0.88	0.79	0.71	0.69	0.64
	dihomo-linolenate (20:3n3 or n6)	1.00	0.97	0.93	0.83	0.75	0.74
Fatty Acid, Dicarboxylate	2-hydroxyadipate	0.79	0.70	0.65	0.60	0.57	0.71
	suberate (octanedioate)	0.81	0.70	0.77	0.65	0.60	0.79
	sebacate (decanedioate)	0.82	0.73	0.79	0.65	0.62	0.76
	1,11-undecanedicarboxylate	0.77	0.69	0.75	0.68	0.66	0.91
	tetradecanedioate	0.90	0.81	0.83	0.80	0.75	0.84
	hexadecanedioate	0.77	0.69	0.72	0.70	0.69	0.74
	docosadioate	0.84	0.81	0.83	0.70	0.76	0.72
	3-carboxy-4-methyl-5-propyl-2-furanpropanoate (CMPF)	0.82	0.81	0.72	0.56	0.63	0.82
Fatty Acid Metabolism (also BCAA Metabolism)	butyrylcarnitine	0.85	0.78	0.80	0.81	0.80	0.69
	propionylcarnitine	0.65	0.63	0.59	0.67	0.63	0.62
Fatty Acid, Monohydroxy	2-hydroxyoctanoate	0.81	0.79	0.87	0.71	0.55	0.48
	2-hydroxydecanoate	0.86	0.77	0.86	0.67	0.47	0.41
	3-hydroxysebacate	0.58	0.60	0.49	0.46	0.47	0.64
	3-hydroxylaurate	0.74	0.71	0.75	0.64	0.64	0.64
	3-hydroxymyristate	0.86	0.82	0.85	0.69	0.77	0.70
	5-hydroxyhexanoate	0.94	0.82	0.78	0.71	0.64	0.83
	5-hydroxydecanoate	0.84	0.69	0.76	0.67	0.58	0.78
	16-hydroxypalmitate	0.90	0.78	0.81	0.80	0.73	0.69
Endocannabinoid	N-palmitoyltaurine	1.02	0.96	0.96	0.82	0.75	0.76
Inositol Metabolism	myo-inositol	0.66	0.66	0.76	0.77	0.81	0.74
	scyllo-inositol	0.60	0.59	0.70	0.79	0.78	0.65
	inositol 1-phosphate (I1P)	0.73	0.82	0.71	0.75	0.68	0.58
Lysolipid	2-palmitoleoylglycerophosphocholine	0.83	0.78	0.61	0.61	0.38	0.54
	1-palmitoylplasmenylethanolamine	0.66	0.72	0.65	0.67	0.54	0.50
	1-oleoylplasmenylethanolamine	0.57	0.74	0.62	0.52	0.35	0.32
	1-oleoylglycerophosphoethanolamine	0.74	0.65	0.53	0.47	0.54	0.60
	1-linoleoylglycerophosphoethanolamine	0.78	0.75	0.65	0.60	0.70	0.70
	1-arachidonoylglycerophosphoethanolamine	0.86	0.82	0.75	0.69	0.76	0.73
	1-oleoylglycerophosphoinositol	0.93	1.24	1.05	0.30	0.31	0.26
	1-linoleoylglycerophosphoinositol	1.13	1.17	0.88	0.65	0.65	0.72
	1-linoleoylglycerophosphoserine	0.76	0.80	0.28	0.22	0.23	0.12
	1-arachidonoylglyercophosphate	1.11	0.84	0.41	0.61	0.36	0.37
	oleoyl-linoleoyl-glycerophosphoinositol	0.83	0.84	0.80	0.58	0.59	0.56
	palmitoyl-linoleoyl-glycerophosphoinositol	0.82	0.88	0.84	0.63	0.59	0.58
Glycerolipid Metabolism	glycerol 3-phosphate (G3P)	0.59	0.52	0.70	0.67	0.77	0.68
	glycerophosphoglycerol	0.72	0.62	0.60	0.61	0.60	0.60
Glycerolipid Metabolism	sphingosine	0.53	0.41	0.34	0.23	0.36	0.16
Mevalonate Metabolism	mevalonate	1.11	0.95	0.84	0.68	0.65	0.59
Sterol	beta-sitosterol	0.88	0.94	0.80	0.74	0.79	0.79
	campesterol	0.85	0.89	0.81	0.80	0.77	0.80
	fucosterol	0.94	0.91	0.81	0.65	0.72	0.61
Steroid	cortisol	0.38	0.33	0.44	0.84	0.67	0.69
Primary Bile Acid Metabolism	cholate	0.03	0.01	0.01	0.00	0.01	0.01
	taurocholate	0.14	0.19	0.17	0.19	0.19	0.19
Secondary Bile Acid Metabolism	deoxycholate	0.38	0.41	0.35	0.26	0.32	0.29
	ursodeoxycholate	0.55	0.60	0.51	0.24	0.31	0.36

Numbers in green text were significantly lower than the baseline (wk 0).

**Table 3:** Serum metabolites related to amino acid and peptide metabolism that were increased in cats undergoing weight loss.

Metabolic Pathway	Metabolite	Fold Change					
		wk 1	wk 2	wk 4	wk 8	wk 12	wk 16
		wk 0	wk 0	wk 0	wk 0	wk 0	wk 0
*Amino Acids*							
Glycine, Serine and Threonine Metabolism	N-acetylglycine	1.37	1.41	1.51	1.61	1.66	1.63
Glutamate Metabolism	glutamine	1.05	1.10	1.09	1.10	1.19	1.06
Histidine Metabolism	1-methylhistidine	1.20	1.20	1.17	1.28	1.34	1.28
Lysine Metabolism	N6-acetyllysine	1.01	1.08	1.15	1.19	1.15	1.17
	N-6-trimethyllysine	1.16	1.21	1.19	1.25	1.22	1.24
	glutarylcarnitine (C5)	1.19	1.32	1.43	1.46	1.53	1.47
	3-methylglutarylcarnitine (1)	1.41	1.43	1.68	1.95	2.2	1.74
Phenylalanine and Tyrosine Metabolism	3-(4-hydroxyphenyl)lactate	1.09	1.09	1.15	1.16	1.17	1.09
Tryptophan Metabolism	kynurenine	1.04	1.05	1.16	1.18	1.19	1.19
	tryptophan betaine	1.03	1.02	1.14	1.29	1.77	2.25
Leucine, Isoleucine and Valine Metabolism	isovalerylglycine	1.21	1.08	1.18	1.28	1.41	1.38
	3-hydroxy-2-ethylpropionate	1.32	1.19	1.30	1.63	1.53	1.48
	6-hydroxynorleucine	1.10	1.14	1.18	1.24	1.25	1.15
Methionine, Cysteine, SAM and Taurine Metabolism	N-formylmethionine	1.04	1.06	1.07	1.10	1.11	1.10
	2-aminobutyrate	1.23	1.20	1.29	1.50	1.41	1.28
Urea cycle; Arginine and Proline Metabolism	pro-hydroxy-pro	1.16	1.47	1.63	1.55	1.50	1.92
Glutathione Metabolism	ophthalmate	0.99	1.20	2.02	3.68	3.09	2.64
Felinine Metabolism	felinine	1.07	1.00	1.04	1.06	1.11	1.08
*Peptides*							
Gamma-glutamyl Amino Acid	gamma-glutamylalanine	1.07	1.03	1.24	1.36	1.37	1.28
	gamma-glutamylglutamine	1.12	1.12	1.16	1.25	1.20	1.18
	gamma-glutamylisoleucine	1.23	1.22	1.23	1.35	1.22	1.25
	gamma-glutamylleucine	1.16	1.14	1.17	1.19	1.14	1.15
	gamma-glutamylvaline	1.20	1.12	1.15	1.24	1.19	1.14
	gamma-glutamyl-2-aminobutyrate	1.41	1.56	1.77	2.11	1.99	1.91
Dipeptide Derivative	N-acetylcarnosine	1.24	1.33	1.37	1.37	1.27	1.36
	anserine	1.08	1.09	1.11	1.16	1.22	1.17
Dipeptide	prolylglycine	1.27	1.35	1.34	1.49	1.38	1.33

Numbers in red text were significantly higher than the baseline (wk 0).

**Table 4:** Serum metabolites related to amino acid metabolism that were decreased in cats undergoing weight loss.

Metabolic Pathway	Metabolite	Fold Change					
		wk 1	wk 2	wk 4	wk 8	wk 12	wk 16
		wk 0	wk 0	wk 0	wk 0	wk 0	wk 0
Amino Acids							
Glycine, Serine and Threonine Metabolism	sarcosine (N-Methylglycine)	0.62	0.72	0.61	0.50	0.57	0.53
	threonine	0.95	0.92	0.87	0.82	0.78	0.80
Glutamate Metabolism	glutamate	0.71	0.73	0.82	0.81	0.95	0.67
Lysine Metabolism	glutarate (pentanedioate)	0.75	0.64	0.67	0.61	0.63	0.71
Phenylalanine and Tyrosine Metabolism	N-acetylphenylalanine	0.87	0.81	0.81	0.80	0.77	0.80
	phenyllactate (PLA)	0.70	0.68	0.72	0.74	0.74	0.69
	o-cresol sulfate	0.68	0.49	0.26	0.27	0.36	0.47
	Gentisate	0.79	0.85	0.58	0.72	0.45	0.59
	3-[3-(sulfooxy)phenyl]propanoic acid	0.79	0.86	0.47	0.56	0.47	0.47
	3-(3-hydroxyphenyl)propionate	0.68	0.75	0.45	0.52	0.45	0.48
	3-(4-hydroxyphenyl)propionate	0.45	0.57	0.37	0.32	0.31	0.28
	4-hydroxycinnamate sulfate	0.66	0.69	0.43	0.38	0.31	0.28
Tryptophan Metabolism	N-acetyltryptophan	0.85	0.78	0.76	0.65	0.68	0.70
	indolelactate	0.87	0.87	0.91	0.89	0.87	0.83
	indolepropionate	0.71	0.66	0.64	0.59	0.61	0.56
	picolinate	0.95	0.89	0.72	0.58	0.58	0.48
	indole-3-carboxylic acid	0.82	0.79	0.78	0.53	0.49	0.49
Leucine, Isoleucine and Valine Metabolism	leucine	1.02	1.00	0.98	0.93	0.89	0.95
	2-hydroxy-3-methylvalerate	0.57	0.55	0.60	0.65	0.70	0.66
Methionine, Cysteine, SAM and Taurine Metabolism	methionine	0.91	0.88	0.85	0.76	0.71	0.79
	N-acetylmethionine	0.85	0.83	0.79	0.83	0.80	0.80
	methionine sulfoxide	0.73	0.74	0.71	0.60	0.60	0.69
	S-adenosylhomocysteine (SAH)	0.63	0.56	0.63	0.72	0.46	0.50
	Cystathionine	0.80	0.80	0.79	0.68	0.76	0.76
	hypotaurine	0.31	0.31	0.35	0.41	0.36	0.28
	taurine	0.72	0.72	0.72	0.72	0.68	0.62
	N-acetyltaurine	0.86	0.86	0.82	0.81	0.84	0.74
Urea cycle; Arginine and Proline Metabolism	urea	1.05	0.95	0.93	0.83	0.83	0.79
	citrulline	0.96	0.94	0.98	0.91	0.87	0.85
	N-delta-acetylornithine	0.94	0.89	0.86	0.81	0.77	0.74
	N-methylproline	0.86	0.88	0.80	0.80	0.74	0.80

Numbers in green text were significantly lower than the baseline (wk 0).

**Table 5:** Altered serum metabolites related to carbohydrate and energy metabolism in cats undergoing weight loss.

Metabolic Pathway	Metabolite	Fold Change					
		wk 1	wk 2	wk 4	wk 8	wk 12	wk 16
		wk 0	wk 0	wk 0	wk 0	wk 0	wk 0
*Carbohydrates*							
Pentose Metabolism	ribose	0.68	0.52	0.48	0.59	0.73	0.59
Aminosugar Metabolism	glucuronate	1.03	0.98	0.93	0.90	0.91	0.85
	N-acetylneuraminate	0.74	0.72	0.61	0.62	0.56	0.54
*Energy*							
TCA Cycle	citrate	1.09	1.07	0.99	0.98	1.05	1.09
	succinylcarnitine	1.18	1.23	1.33	1.51	1.65	1.53
	alpha-ketoglutarate	0.83	0.81	0.85	0.94	0.92	0.95
	succinate	0.81	0.83	0.84	0.91	0.91	0.83
	fumarate	0.56	0.54	0.59	0.75	0.70	0.69
Oxidative Phosphorylation	phosphate	0.93	0.93	0.91	0.92	0.90	0.88

Numbers in red text were significantly higher, while numbers in green text were significantly lower than the baseline (wk 0).

**Table 6:** Serum metabolites related to nucleotide, xenobiotic, and cofactor and vitamin metabolism that were increased in cats undergoing weight loss.

Metabolic Pathway	Metabolite	Fold Change					
		wk 1	wk 2	wk 4	wk 8	wk 12	wk 16
		wk 0	wk 0	wk 0	wk 0	wk 0	wk 0
*Nucleotide*							
Purine Metabolism, Adenine containing	N6-methyladenosine	1.10	1.33	1.60	1.66	1.62	1.63
	N6-carbamoylthreonyladenosine	1.02	1.12	1.27	1.36	1.30	1.22
	7-methylguanine	1.08	1.06	1.07	1.06	1.11	1.12
Pyrimidine Metabolism, Orotate containing	orotate	1.10	1.08	1.17	1.26	1.38	1.20
	N4-acetylcytidine	1.13	1.11	1.27	1.31	1.21	1.22
Pyrimidine Metabolism, Thymine containing	3-aminoisobutyrate	1.11	1.25	1.13	1.29	1.19	1.18
*Xenobiotics*							
Food Component/Plant	4-allylphenol sulfate	1.05	1.06	1.14	1.28	1.32	1.44
Chemical	2-hydroxyisobutyrate	1.18	1.27	1.34	1.63	1.9	1.87
*Vitamins and Cofactors*							
Tocopherol Metabolism	alpha-CEHC sulfate	1.07	1.06	1.09	1.44	1.44	1.53

Numbers in red text were significantly higher than the baseline (wk 0).

**Table 7:** Serum metabolites related to nucleotide, xenobiotic, and cofactor and vitamin metabolism that were decreased in cats undergoing weight loss.

Metabolic Pathway	Metabolite	Fold Change					
		wk 1	wk 2	wk 4	wk 8	wk 12	wk 16
		wk 0	wk 0	wk 0	wk 0	wk 0	wk 0
*Nucleotide*							
Purine Metabolism, (Hypo)Xanthine/ Inosine containing	hypoxanthine	0.81	0.74	0.72	0.78	0.80	0.70
	xanthine	0.48	0.44	0.51	0.66	0.63	0.70
	2’-deoxyinosine	0.46	0.51	0.45	0.55	0.74	0.63
	urate	0.74	0.70	0.77	0.84	0.74	0.84
Purine Metabolism, Guanine containing	guanine	0.68	0.58	0.49	0.65	0.62	0.61
Pyrimidine Metabolism, Uracil containing	2’-deoxyuridine	0.63	0.60	0.68	0.86	0.79	0.75
Pyrimidine Metabolism, Cytidine containing	cytidine	0.89	0.86	0.80	0.79	0.67	0.68
*Xenobiotics*							
Benzoate Metabolism	2-hydroxyhippurate (salicylurate)	0.89	0.93	0.88	0.73	0.64	0.62
	4-hydroxyhippurate	0.87	0.87	0.58	0.58	0.58	0.50
	4-ethylphenylsulfate	0.41	0.46	0.24	0.34	0.26	0.22
	4-vinylphenol sulfate	0.49	0.36	0.31	0.24	0.19	0.19
	3-methoxycatechol sulfate (2)	0.45	0.67	0.38	0.55	0.46	0.46
	methyl-4-hydroxybenzoate sulfate	0.35	0.22	0.14	0.18	0.24	0.49
Food Component/Plant	gluconate	0.64	0.63	0.76	0.71	0.37	0.34
	equol sulfate	0.67	0.84	0.47	0.41	0.17	0.07
	ergothioneine	0.85	0.80	0.79	0.74	0.64	0.57
	ferulic acid 4-sulfate	0.37	0.65	0.35	0.08	0.14	0.23
	indoleacrylate	0.81	0.85	0.84	0.70	0.72	0.73
	thymol sulfate	0.72	0.57	0.41	0.22	0.14	0.10
	methyl glucopyranoside (alpha + beta)	0.93	0.92	0.80	0.63	0.55	0.39
	pyrraline	0.82	0.83	0.72	0.60	0.72	0.78
Drug	4-acetylphenol sulfate	0.40	0.44	0.44	0.26	0.89	0.29
	hydroquinone sulfate	0.70	0.78	0.46	0.47	0.44	0.51
	salicylate	0.73	0.71	0.65	0.50	0.34	0.31
Chemical	O-sulfo-l-tyrosine	0.84	0.8	0.76	0.77	0.80	0.69
	ethyl glucuronide	0.17	0.24	0.31	0.28	0.91	1.36
	2-aminophenol sulfate	0.83	0.91	0.67	0.63	0.54	0.60
	3-hydroxypyridine sulfate	0.69	0.74	0.54	0.45	0.65	0.78
*Vitamins and Cofactors*							
Nicotinate and Nicotinamide Metabolism	nicotinamide	0.43	0.44	0.61	0.62	0.57	0.55
Riboflavin Metabolism	riboflavin (Vitamin B2)	0.95	0.81	0.75	0.69	0.61	0.77
Tocopherol Metabolism	gamma-tocopherol	0.82	0.89	0.76	0.64	0.55	0.56
Vitamin B6 Metabolism	pyridoxine (Vitamin B6)	0.72	0.74	0.69	0.74	0.54	0.46

Numbers in green text were significantly lower than the baseline (wk 0).

## Data Availability

The data presented in this study are available on request from the corresponding author.

## Supplementary Materials

The following are available online at https://www.mdpi.com/article/10.3390/metabo11050324/s1; Table S1: Serum metabolites related to lipid metabolism that were altered in cats undergoing weight loss; Table S2: Serum metabolites related to amino acid and peptide metabolism that were altered in cats undergoing weight loss; Table S3: Serum metabolites related to carbohydrate and energy metabolism that were altered in cats undergoing weight loss; Table S4: Serum metabolites related to nucleotide, xenobiotic, and cofactor and vitamin metabolism that were altered in cats undergoing weight loss; Table S5: Correlation coefficients (r) between fasted serum chemistry measures and metabolites; Table S6: Correlation coefficients (r) between DEXA scan results and metabolites.

## Abbreviations

AA	amino acid
ANOVA	analysis of variance
BCAA	branched-chain amino acids
BCS	body condition score
BHBA	3-hydroxybutyrate
BUN	blood urea nitrogen
BW	body weight
DiHOME	dihydroxyoctadecanoic acid
DM	diabetes mellitus
EpOME	epoxy octadecenoic acid
FA	fatty acids
G3P	glycerol 3-phosphate
GC	gas chromatography
HETE	12-hydroxyeicosatetraenoic acid
HFD	high-fat diet
HMGR	HMG-CoA reductase
LC	liquid chromatography
LDL	low-density lipoprotein
LSD	least significant difference
MDA	mean decrease accuracy
ME	metabolizable energy
MER	maintenance energy requirement
MS	mass spectral
NEFA	non-esterified fatty acids
OOB	out-of-bag
PC	phosphatidylcholine
PCA	principal component analysis
PUFA	polyunsaturated fatty acids
QC	quality control
RF	random forest
RI	retention index
RSD	relative standard deviation
SAH	s-adenosylhomocysteine
SAM	s-adenosylmethionine
SFA	saturated fatty acids
TCA	tricarboxylic acid cycle
TGRL	triglyceride-rich lipoprotein
VLDL	very-low-density lipoprotein
WK	week
