# Supplementary material for: Effects of Weight Loss and Moderate-Protein, High-Fiber Diet Consumption on the Fasted Serum Metabolome of Cats
Source: Metabolites. 2021 May 18;11(5):324. doi: 10.3390/metabo11050324 (PMC8158395; doi:10.3390/metabo11050324)
Supplement: Supplementary file 1 [file metabolites-11-00324-s001.zip › metabolites-1199304-supplementary.pdf]

Supplementary tables

Table S1. Serum metabolites related to lipid metabolism that were altered in cats undergoing weight loss[a,b]

Metabolic Pathway	Metabolite	Fold change wk 1 / wk 0	wk 2 / wk 0	wk 4 / wk 0	wk 8 / wk 0	wk 12 / wk 0	wk 16 / wk 0
Lipids							
Medium Chain Fatty Acid	heptanoate (7:0)	0.89	0.86	0.94	0.9	**0.85**	**0.84**
	pelargonate (9:0)	0.83	**0.75**	0.84	**0.72**	**0.67**	**1.26**
	caprate (10:0)	0.91	**0.88**	0.92	**0.88**	**0.79**	0.96
	undecanoate (11:0)	0.86	0.83	0.86	0.94	**0.73**	0.99
	10-undecenoate (11:1n1)	**1.11**	**1.21**	**1.4**	**1.52**	**1.48**	**1.6**
	laurate (12:0)	0.95	0.85	0.88	0.89	**0.73**	**0.74**
Long Chain Fatty Acid	pentadecanoate (15:0)	0.93	0.9	0.85	**0.8**	**0.8**	**0.81**
	palmitate (16:0)	1	0.99	0.96	**0.87**	**0.87**	**0.9**
	palmitoleate (16:1n7)	0.98	0.94	0.9	0.88	0.86	**0.82**
	margarate (17:0)	0.97	0.98	0.88	**0.76**	**0.81**	**0.77**
	10-heptadecenoate (17:1n7)	0.99	0.92	0.85	**0.81**	0.82	**0.74**
	stearate (18:0)	1.02	1.03	0.96	**0.85**	**0.88**	**0.87**
	oleate (18:1n9)	0.88	0.95	0.86	**0.8**	0.89	**0.82**
	cis-vaccenate (18:1n7)	0.93	1.06	0.89	**0.85**	0.91	0.86
	arachidate (20:0)	1.04	1.06	0.97	**0.82**	**0.86**	**0.86**
Polyunsaturated Fatty Acid (n3 and n6)	stearidonate (18:4n3)	0.91	0.87	0.81	**0.66**	**0.55**	**0.49**
	eicosapentaenoate (EPA; 20:5n3)	0.95	0.94	0.86	**0.7**	**0.65**	**0.74**
	docosahexaenoate (DHA; 22:6n3)	1.15	**1.16**	1.14	1.08	0.94	0.98
	linoleate (18:2n6)	0.96	0.96	0.88	**0.83**	**0.82**	**0.85**
	linolenate (18:3n3 or 6)	0.88	0.88	**0.79**	**0.71**	**0.69**	**0.64**
	dihomo-linolenate (20:3n3 or n6)	1	0.97	0.93	**0.83**	**0.75**	**0.74**
	arachidonate (20:4n6)	1.03	1.13	1.13	1.01	0.84	**0.81**
	docosapentaenoate (n6 DPA; 22:5n6)	**1.28**	**1.3**	1.29	1.25	1.13	1.05
Fatty Acid, Branched	15-methylpalmitate (isobar with 2-methylpalmitate)	0.99	1.02	0.88	0.81	0.84	**0.64**
	pristanate	0.96	0.88	0.97	0.88	0.8	**0.68**
Fatty Acid, Dicarboxylate	adipate	0.77	0.71	0.8	**0.67**	**0.71**	0.74
	2-hydroxyadipate	**0.79**	**0.7**	**0.65**	**0.6**	**0.57**	**0.71**
	maleate (cis-Butenedioate)	0.93	0.93	**0.83**	**0.78**	0.92	**0.85**
	pimelate (heptanedioate)	0.82	**0.73**	**0.76**	**0.66**	**0.63**	0.83
	suberate (octanedioate)	**0.81**	**0.7**	**0.77**	**0.65**	**0.6**	**0.79**
	azelate (nonanedioate)	**0.81**	**0.7**	0.78	**0.65**	**0.6**	0.84
	sebacate (decanedioate)	0.82	**0.73**	0.79	**0.65**	**0.62**	**0.76**
	undecanedioate	0.83	**0.73**	0.79	**0.68**	**0.65**	0.83
	1,11-undecanedicarboxylate	**0.77**	**0.69**	**0.75**	**0.68**	**0.66**	0.91
	dodecanedioate	0.84	**0.75**	0.78	**0.69**	**0.67**	0.84
	tetradecanedioate	0.9	**0.81**	**0.83**	**0.8**	**0.75**	**0.84**
	hexadecanedioate	**0.77**	**0.69**	**0.72**	**0.7**	**0.69**	**0.74**
	octadecanedioate	0.9	**0.9**	0.98	**0.91**	0.98	0.97

Table S1 (cont.)

	eicosanodioate	1.02	1.03	1.12	1.09	**1.16**	1.09
	docosadioate	0.84	**0.81**	**0.83**	**0.7**	**0.76**	**0.72**
	3-carboxy-4-methyl-5-propyl-2-furanpropanoate (CMPF)	**0.82**	**0.81**	**0.72**	**0.56**	**0.63**	**0.82**
Fatty Acid, Amino	2-aminoheptanoate	0.96	1.05	1.09	1.14	1.13	**1.26**
	2-aminooctanoate	**1.16**	1.12	**1.21**	1.07	0.95	**0.82**
Fatty Acid Synthesis	malonylcarnitine	1.21	**1.35**	**1.57**	**1.77**	**1.84**	**1.58**
	malonate (propanedioate)	**1.36**	**1.44**	**1.38**	**1.3**	**1.37**	**1.36**
	2-methylmalonyl carnitine	1.22	1.4	**1.68**	**1.96**	**2.14**	**1.86**
Fatty Acid Metabolism (also BCAA Metabolism)	butyrylcarnitine	0.85	**0.78**	**0.8**	**0.81**	**0.8**	**0.69**
	propionylcarnitine	**0.65**	**0.63**	**0.59**	**0.67**	**0.63**	**0.62**
	propionylglycine	0.82	0.85	0.84	0.74	**0.65**	**1.61**
FA Metabolism (Acyl Glycine)	hexanoylglycine	**1.55**	**1.56**	**1.98**	**2.22**	**2.33**	**2.11**
	N-octanoylglycine	1.22	1.3	1.55	**3.04**	**2.78**	1.7
FA Metabolism (Acyl Carnitine)	hydroxybutyrylcarnitine*	0.94	1.13	1.32	**1.85**	**2**	1.63
Ketone Bodies	acetoacetate	**1.54**	**1.59**	**1.93**	**1.91**	**1.54**	1.08
	3-hydroxybutyrate (BHBA)	1.51	**1.72**	**1.95**	**2.09**	**2**	**1.74**
Fatty Acid, Monohydroxy	2-hydroxyoctanoate	**0.81**	**0.79**	**0.87**	**0.71**	**0.55**	**0.48**
	2-hydroxydecanoate	**0.86**	**0.77**	**0.86**	**0.67**	**0.47**	**0.41**
	3-hydroxyoctanoate	1.02	1.06	1.27	1.31	**1.36**	1.32
	3-hydroxydecanoate	0.86	**0.81**	0.94	0.86	**0.83**	**0.86**
	3-hydroxysebacate	**0.58**	**0.6**	**0.49**	**0.46**	**0.47**	**0.64**
	3-hydroxylaurate	**0.74**	**0.71**	**0.75**	**0.64**	**0.64**	**0.64**
	3-hydroxymyristate	0.86	0.82	0.85	**0.69**	**0.77**	**0.7**
	5-hydroxyhexanoate	0.94	**0.82**	**0.78**	**0.71**	**0.64**	**0.83**
	5-hydroxydecanoate	0.84	**0.69**	**0.76**	**0.67**	**0.58**	**0.78**
	8-hydroxyoctanoate	0.8	**0.78**	0.83	**0.7**	**0.67**	0.81
	16-hydroxypalmitate	0.9	**0.78**	**0.81**	**0.8**	**0.73**	**0.69**
	13-HODE + 9-HODE	0.81	0.78	**0.62**	**0.59**	**0.47**	**0.35**
Fatty Acid, Dihydroxy	12,13-DiHOME	**0.74**	**0.62**	**0.6**	**0.44**	**0.3**	**0.24**
	9,10-DiHOME	0.86	0.74	0.73	**0.48**	**0.42**	**0.38**
Eicosanoid	thromboxane B2	0.68	1.23	0.81	0.82	0.48	**0.31**
	12-HETE	**0.56**	0.82	**0.61**	**0.54**	**0.4**	**0.25**
Endocannabinoid	oleic ethanolamide	1.18	**1.31**	1.19	1.05	1.01	1.03
	palmitoyl ethanolamide	1.17	1.21	1.23	**1.22**	1.15	**1.28**
	N-stearoyltaurine	1.11	1.05	0.99	0.91	**0.77**	**0.7**
	N-palmitoyltaurine	1.02	0.96	0.96	**0.82**	**0.75**	**0.76**
	N-linolenoyltaurine *	0.8	1.2	0.99	0.77	0.82	**0.58**
Inositol Metabolism	myo-inositol	**0.66**	**0.66**	**0.76**	**0.77**	**0.81**	**0.74**
	scyllo-inositol	**0.6**	**0.59**	**0.7**	**0.79**	**0.78**	**0.65**
	inositol 1-phosphate (I1P)	**0.73**	**0.82**	**0.71**	**0.75**	**0.68**	**0.58**

Table S1 (cont.)

Phospholipid Metabolism	choline	0.73	0.69	0.71	0.69	0.7	**0.59**
	choline phosphate	**0.63**	**0.64**	**0.63**	**0.64**	**0.59**	**0.53**
	glycerophosphorylcholine (GPC)	**0.72**	**0.73**	**0.64**	**0.62**	**0.66**	**0.7**
	phosphoethanolamine	**0.51**	**0.54**	**0.64**	**0.64**	**0.61**	**0.51**
	glycerophosphoethanolamine	**0.67**	**0.64**	**0.57**	**0.59**	**0.65**	**0.59**
Lysolipid	1-palmitoylglycerophosphocholine	1.19	0.7	0.68	0.99	0.89	**0.38**
	1-palmitoleoylglycerophosphocholine	1.46	0.75	0.7	1.57	1	**0.35**
	2-palmitoleoylglycerophosphocholine	0.83	**0.78**	**0.61**	**0.61**	**0.38**	**0.54**
	1-stearoylglycerophosphocholine	1.01	0.55	0.56	0.91	0.75	**0.32**
	2-stearoylglycerophosphocholine	1.42	0.62	0.53	1.11	0.59	**0.3**
	1-oleoylglycerophosphocholine	1.18	0.66	0.55	0.97	0.72	**0.31**
	1-linoleoylglycerophosphocholine	1.16	0.73	0.62	0.98	0.82	**0.35**
	1-linolenoylglycerophosphocholine	1.18	0.6	0.47	0.78	0.58	**0.17**
	1-palmitoylplasmenylethanolamine	0.66	0.72	**0.65**	**0.67**	**0.54**	**0.5**
	1-stearoylplasmenylethanolamine	0.85	1.3	1.26	0.96	0.98	**0.56**
	1-oleoylplasmenylethanolamine	0.57	0.74	**0.62**	**0.52**	**0.35**	**0.32**
	1-oleoylglycerophosphoethanolamine	**0.74**	**0.65**	**0.53**	**0.47**	**0.54**	**0.6**
	1-linoleoylglycerophosphoethanolamine	0.78	0.75	**0.65**	**0.6**	**0.7**	**0.7**
	1-arachidonoylglycerophosphoethanolamine	0.86	0.82	0.75	**0.69**	**0.76**	**0.73**
	1-palmitoylglycerophosphoinositol	1.56	**1.73**	1.37	1.07	**0.32**	0.82
	1-stearoylglycerophosphoinositol	1.1	1.19	1.05	0.73	**0.6**	**0.6**
	1-oleoylglycerophosphoinositol	0.93	1.24	1.05	**0.3**	**0.31**	**0.26**
	1-linoleoylglycerophosphoinositol	1.13	1.17	0.88	**0.65**	**0.65**	**0.72**
	1-arachidonoylglycerophosphoinositol	**1.29**	**1.4**	1.12	0.95	0.83	0.98
	1-linoleoylglycerophosphoserine	0.76	0.8	**0.28**	**0.22**	**0.23**	**0.12**
	1-arachidonoylglyercophosphate	1.11	0.84	**0.41**	**0.61**	**0.36**	**0.37**
	oleoyl-linoleoyl-glycerophosphoinositol	0.83	**0.84**	**0.8**	**0.58**	**0.59**	**0.56**
	palmitoyl-arachidonoyl-glycerophosphocholine	1.3	1.33	1.06	1.17	1.08	**1.36**
	palmitoyl-linoleoyl-glycerophosphoinositol	0.82	0.88	0.84	**0.63**	**0.59**	**0.58**
	stearoyl-arachidonoyl-glycerophosphocholine	1.01	**1.14**	**1.15**	1.04	0.96	**0.89**
	stearoyl-arachidonoyl-glycerophosphoinositol	0.98	0.96	1	0.85	**0.78**	**0.75**
	stearoyl-linoleoyl-glycerophosphocholine	0.97	0.95	0.85	**0.8**	**0.81**	0.94
	stearoyl-arachidonoyl-glycerophosphoinositol	1.79	1.14	1.47	**2.03**	1.76	**2.78**
Glycerolipid Metabolism	glycerol	0.9	**0.78**	1	1.08	1	0.99
	glycerol 3-phosphate (G3P)	**0.59**	**0.52**	0.7	**0.67**	0.77	**0.68**
	glycerophosphoglycerol	**0.72**	**0.62**	**0.6**	**0.61**	**0.6**	**0.6**

Table S1 (cont.)

Monoacylglycerol	1-palmitoylglycerol (1-monopalmitin)	**1.41**	**1.5**	**1.69**	**1.85**	**1.68**	**1.74**
	2-palmitoylglycerol (2-monopalmitin)	**3.05**	1.81	**2.18**	**2.74**	**2.59**	**1.92**
	1-stearoylglycerol (1-monostearin)	0.99	1.07	1.19	1.1	**1.22**	1.06
	1-linoleoylglycerol (1-monolinolein)	**1.37**	**1.65**	**1.81**	**1.59**	**1.45**	**1.65**
	2-linoleoylglycerol (2-monolinolein)	1.48	1.76	**2.86**	**2.17**	1.47	2.05
	1-arachidonylglycerol	**1.79**	**2.35**	**2.73**	**2.45**	**2.18**	**2.41**
	2-arachidonoyl glycerol	1.48	2.91	**3.07**	**2.61**	2.33	2.19
	1-docosahexaenoylglycerol	**1.52**	**2.31**	**2.53**	**2.15**	**1.76**	**2.26**
	2-docosahexaenoylglcyerol*	1.71	1.67	**2.71**	1.87	1.32	1.34
Sphingolipid Metabolism	stearoyl sphingomyelin	1.07	1.09	1.19	**1.35**	**1.24**	**1.37**
	oleoyl sphingomyelin	1.11	1.13	**1.33**	**1.32**	**1.24**	**1.35**
	sphingosine	0.53	**0.41**	**0.34**	**0.23**	**0.36**	**0.16**
	palmitoleoyl sphingomyelin*	0.99	1.07	**1.19**	1.07	1.11	1.12
	erucoyl sphingomyelin*	1.17	1.17	1.57	1.52	1.35	**1.96**
	arachidoyl sphingomyelin*	2.21	0.9	2	1.81	**2.56**	**2.09**
Mevalonate Metabolism	mevalonate	1.11	0.95	0.84	**0.68**	**0.65**	**0.59**
Sterol	cholesterol	0.96	0.99	0.95	0.94	**0.93**	0.95
	7-alpha-hydroxy-3-oxo-4-cholestenoate (7-Hoca)	0.97	1	1.09	**1.1**	1.07	1.04
	cholestanol	0.9	0.91	0.87	0.86	**0.79**	**0.81**
	beta-sitosterol	0.88	0.94	0.8	**0.74**	**0.79**	**0.79**
	campesterol	0.85	0.89	**0.81**	**0.8**	**0.77**	**0.8**
	fucosterol	0.94	0.91	0.81	**0.65**	**0.72**	**0.61**
Steroid	5alpha-pregnan-3beta,20beta-diol monosulfate (1)	1.18	1.11	1.18	**1.21**	1.12	1.05
	cortisol	**0.38**	**0.33**	**0.44**	0.84	0.67	0.69
	cortisone	**0.6**	**0.48**	0.73	0.93	0.94	0.89
Primary Bile Acid Metabolism	cholate	**0.03**	**0.01**	**0.01**	**0**	**0.01**	**0.01**
	taurocholate	**0.14**	**0.19**	**0.17**	**0.19**	**0.19**	**0.19**
Secondary Bile Acid Metabolism	deoxycholate	**0.38**	**0.41**	**0.35**	**0.26**	**0.32**	**0.29**
	taurolithocholate 3-sulfate	0.97	**0.83**	0.88	0.91	0.94	0.91
	ursodeoxycholate	0.55	0.6	**0.51**	**0.24**	**0.31**	**0.36**

[a]For each metabolite, mean value is the group mean of re-scaled data to have median equal to 1.
[b]Mean values in green were decreased, whereas in red were increased with *P*- and *q*- values < 0.05. P values were calculated from one way Anova; *q*- values were used to estimate the false discovery rate (FDR) in multiple comparisons.

Table S2. Serum metabolites related to amino acid and peptide metabolism that were altered in cats undergoing weight loss[a,b]

Metabolic Pathway	Metabolite	Fold change wk 1 / wk 0	wk 2 / wk 0	wk 4 / wk 0	wk 8 / wk 0	wk 12 / wk 0	wk 16 / wk 0
	Amino Acids						
Glycine, Serine and Threonine Metabolism	glycine	1.1	1.09	1.18	1.22	**1.25**	1.08
	N-acetylglycine	**1.37**	**1.41**	**1.51**	**1.61**	**1.66**	**1.63**
	sarcosine (N-Methylglycine)	**0.62**	**0.72**	**0.61**	**0.5**	**0.57**	**0.53**
	threonine	0.95	**0.92**	**0.87**	**0.82**	**0.78**	**0.8**
	N-acetylthreonine	1.07	1.1	1.11	1.13	**1.16**	1.03
Alanine and Aspartate Metabolism	aspartate	0.76	1	1.51	1.72	**2.32**	1.71
Glutamate Metabolism	glutamate	**0.71**	**0.73**	0.82	0.81	0.95	**0.67**
	glutamine	1.05	1.1	1.09	1.1	**1.19**	1.06
Histidine Metabolism	histidine	0.98	0.94	0.95	0.94	0.95	**0.91**
	1-methylhistidine	**1.2**	**1.2**	**1.17**	**1.28**	**1.34**	**1.28**
	3-methylhistidine	1.14	1.12	1.13	1.12	**1.31**	1.15
	trans-urocanate	0.99	0.81	1.06	**1.85**	1.25	1.14
	imidazole lactate	1.15	1.2	1.33	1.33	**1.37**	1.18
	N-acetylhistamine	0.57	0.75	0.76	0.74	**0.47**	**0.48**
Lysine Metabolism	lysine	1.04	1.04	1.06	**1.1**	1.07	1.04
	N6-acetyllysine	1.01	**1.08**	**1.15**	**1.19**	**1.15**	**1.17**
	N-6-trimethyllysine	**1.16**	**1.21**	**1.19**	**1.25**	**1.22**	**1.24**
	2-aminoadipate	**0.89**	0.92	1.06	**1.19**	**1.15**	**1.12**
	glutarate (pentanedioate)	**0.75**	**0.64**	**0.67**	**0.61**	**0.63**	**0.71**
	glutarylcarnitine (C5)	1.19	**1.32**	**1.43**	**1.46**	**1.53**	**1.47**
	3-methylglutarylcarnitine (1)	**1.41**	**1.43**	**1.68**	**1.95**	**2.2**	**1.74**
	N-acetyl-cadaverine	1.14	1.21	1.01	0.98	0.94	**0.82**
Phenylalanine and Tyrosine Metabolism	phenylalanine	1.01	0.99	1	0.94	**0.89**	**0.92**
	N-acetylphenylalanine	**0.87**	**0.81**	**0.81**	**0.8**	**0.77**	**0.8**
	phenylpyruvate	0.92	1.02	0.98	1.08	**0.8**	1.09
	phenyllactate (PLA)	**0.7**	**0.68**	**0.72**	**0.74**	**0.74**	**0.69**
	4-hydroxyphenylacetate	0.55	0.84	0.89	0.72	**0.41**	**0.21**
	phenylacetylglutamine	0.76	1.12	0.8	0.93	0.72	**0.55**
	tyrosine	1	0.95	1	0.95	**0.88**	**0.87**
	tyramine	0.54	0.95	0.7	0.87	**0.47**	**0.43**
	3-(4-hydroxyphenyl)lactate	**1.09**	**1.09**	**1.15**	**1.16**	**1.17**	**1.09**
	phenol sulfate	1.06	0.93	1.46	**1.51**	1.22	1.27
	o-cresol sulfate	0.68	**0.49**	**0.26**	**0.27**	**0.36**	**0.47**
	3-methoxytyrosine	1	0.94	1.13	1.09	1.16	**1.3**
	Gentisate	0.79	0.85	**0.58**	**0.72**	**0.45**	**0.59**
	3-[3-(sulfooxy)phenyl]propanoic acid	0.79	0.86	**0.47**	**0.56**	**0.47**	**0.47**

Table S2 (cont.)

	3-(3-hydroxyphenyl)propionate	0.68	0.75	**0.45**	**0.52**	**0.45**	**0.48**
	3-(4-hydroxyphenyl)propionate	**0.45**	0.57	**0.37**	**0.32**	**0.31**	**0.28**
	4-hydroxyphenylacetyl glycine	0.67	0.79	0.79	0.63	**0.76**	0.71
	2-hydroxyphenylacetate	0.78	0.93	0.86	**0.8**	1	0.82
	4-hydroxycinnamate sulfate	0.66	0.69	**0.43**	**0.38**	**0.31**	**0.28**
Tryptophan Metabolism	tryptophan	1.06	1.03	**1.1**	1.03	0.99	1.02
	N-acetyltryptophan	0.85	**0.78**	**0.76**	**0.65**	**0.68**	**0.7**
	indolelactate	**0.87**	**0.87**	0.91	**0.89**	**0.87**	**0.83**
	indoleacetate	0.9	0.85	0.93	0.86	0.73	**0.59**
	indolepropionate	**0.71**	**0.66**	**0.64**	**0.59**	**0.61**	**0.56**
	3-indoxyl sulfate	0.92	0.87	**0.7**	0.98	**0.75**	**0.71**
	kynurenine	1.04	1.05	**1.16**	**1.18**	**1.19**	**1.19**
	kynurenate	1.03	1.04	1.1	1.08	**1.21**	1.12
	picolinate	0.95	0.89	0.72	**0.58**	**0.58**	**0.48**
	5-hydroxyindoleacetate	0.87	0.84	0.81	**0.77**	0.94	0.95
	tryptophan betaine	1.03	1.02	1.14	**1.29**	**1.77**	**2.25**
	indole-3-carboxylic acid	0.82	0.79	0.78	**0.53**	**0.49**	**0.49**
	C-glycosyltryptophan	0.94	**0.86**	0.96	0.96	1.01	0.91
Leucine, Isoleucine and Valine Metabolism	leucine	1.02	1	0.98	0.93	**0.89**	0.95
	N-acetylleucine	0.89	0.83	0.82	**0.81**	**0.75**	0.85
	isovalerate	1.02	1.01	0.97	0.91	0.8	**0.67**
	isovalerylglycine	1.21	1.08	1.18	**1.28**	**1.41**	**1.38**
	isovalerylcarnitine	0.79	**0.71**	0.9	**0.77**	0.86	**0.77**
	alpha-hydroxyisovaleroyl carnitine*	0.86	0.84	0.73	0.76	0.64	**0.63**
	alpha-hydroxyisovalerate	0.96	**0.9**	0.96	1.02	**1.11**	1.04
	methylsuccinate	**1.28**	1.15	1.04	1.13	1.08	0.95
	allo-isoleucine	0.97	1.26	1.22	**1.55**	**1.42**	1.18
	3-methyl-2-oxovalerate	1.1	1.04	1.11	1.14	1.1	**1.16**
	2-hydroxy-3-methylvalerate	**0.57**	**0.55**	**0.6**	**0.65**	**0.7**	**0.66**
	3-hydroxy-2-ethylpropionate	**1.32**	1.19	**1.3**	**1.63**	**1.53**	**1.48**
	ethylmalonate	**1.14**	1.09	1.06	0.98	0.93	**0.79**
	isobutyrylglycine	1.24	1.06	0.84	1.24	1.25	**1.51**
	alpha-hydroxyisocaproate	0.94	0.99	1.01	1.06	**1.19**	**1.18**
	6-hydroxynorleucine	1.1	**1.14**	**1.18**	**1.24**	**1.25**	**1.15**
Methionine, Cysteine, SAM and Taurine Metabolism	methionine	0.91	0.88	0.85	**0.76**	**0.71**	**0.79**
	N-acetylmethionine	**0.85**	**0.83**	**0.79**	**0.83**	**0.8**	**0.8**
	N-formylmethionine	1.04	1.06	1.07	**1.1**	**1.11**	**1.1**
	methionine sulfoxide	**0.73**	**0.74**	**0.71**	**0.6**	**0.6**	**0.69**
	S-adenosylhomocysteine (SAH)	**0.63**	**0.56**	**0.63**	**0.72**	**0.46**	**0.5**
	Cystathionine	0.8	0.8	0.79	**0.68**	**0.76**	**0.76**
	2-aminobutyrate	1.23	1.2	**1.29**	**1.5**	**1.41**	**1.28**
	S-methylcysteine	1.05	**1.14**	1.1	1.08	**1.18**	**1.17**
	hypotaurine	**0.31**	**0.31**	**0.35**	**0.41**	**0.36**	**0.28**

Table S2 (cont.)

	taurine	**0.72**	**0.72**	**0.72**	**0.72**	**0.68**	**0.62**
	N-acetyltaurine	**0.86**	**0.86**	**0.82**	**0.81**	**0.84**	**0.74**
Urea cycle; Arginine and Proline Metabolism	urea	1.05	0.95	0.93	**0.83**	**0.83**	**0.79**
	proline	0.91	0.93	0.95	0.92	**0.91**	0.97
	citrulline	0.96	0.94	0.98	**0.91**	**0.87**	**0.85**
	homocitrulline	**1.15**	1.11	**1.11**	**1.12**	1.07	0.98
	dimethylarginine (SDMA + ADMA)	1.14	1.18	1.25	**1.3**	**1.39**	1.13
	N-delta-acetylornithine	0.94	0.89	**0.86**	**0.81**	**0.77**	**0.74**
	N-methylproline	0.86	0.88	**0.8**	**0.8**	**0.74**	**0.8**
	trans-4-hydroxyproline	0.91	1.03	0.97	0.86	1.01	**1.09**
	pro-hydroxy-pro	1.16	**1.47**	**1.63**	**1.55**	**1.5**	**1.92**
Creatine Metabolism	creatine	**0.68**	**0.66**	0.8	0.84	0.85	**0.71**
	creatinine	**1.1**	**1.1**	**1.13**	**1.18**	**1.19**	**1.17**
	creatine phosphate	**0.46**	**0.55**	0.68	**0.72**	0.75	0.64
	guanidinoacetate	0.94	1.07	**1.29**	**1.35**	**1.34**	**1.24**
Glutathione Metabolism	5-methylthioadenosine (MTA)	**0.66**	**0.6**	0.82	0.8	**0.67**	**0.58**
	N-acetylputrescine	0.97	1.02	1.06	0.98	0.94	**0.9**
	cysteine-glutathione disulfide	0.88	0.91	1	**1.11**	1.07	0.99
	ophthalmate	0.99	1.2	**2.02**	**3.68**	**3.09**	**2.64**
Felinine Metabolism	felinine	1.07	1	1.04	**1.06**	**1.11**	**1.08**
	N-acetylfelinine*	**1.16**	1.08	1.03	1.04	0.98	0.98
Peptides							
Gamma-glutamyl Amino Acid	gamma-glutamylalanine	1.07	1.03	**1.24**	**1.36**	**1.37**	**1.28**
	gamma-glutamylglutamate	**0.73**	**0.78**	0.95	1.03	0.98	0.88
	gamma-glutamylglutamine	**1.12**	**1.12**	**1.16**	**1.25**	**1.2**	**1.18**
	gamma-glutamylisoleucine*	**1.23**	**1.22**	**1.23**	**1.35**	**1.22**	**1.25**
	gamma-glutamylleucine	**1.16**	1.14	**1.17**	**1.19**	**1.14**	**1.15**
	gamma-glutamylmethionine	1.02	0.99	0.98	0.92	**0.88**	**0.87**
	gamma-glutamylvaline	**1.2**	1.12	**1.15**	**1.24**	**1.19**	**1.14**
	gamma-glutamyl-2-aminobutyrate	**1.41**	**1.56**	**1.77**	**2.11**	**1.99**	**1.91**
Dipeptide Derivative	carnosine	1.03	0.99	0.95	0.93	0.95	**0.85**
	N-acetylcarnosine	**1.24**	**1.33**	**1.37**	**1.37**	**1.27**	**1.36**
	anserine	1.08	1.09	**1.11**	**1.16**	**1.22**	**1.17**
Dipeptide	glycylleucine	0.73	0.73	0.81	0.73	**0.52**	0.79
	prolylglycine	**1.27**	**1.35**	**1.34**	**1.49**	**1.38**	**1.33**
	valylglycine	0.8	1.18	0.96	0.86	0.85	**0.67**

[a]For each metabolite, mean value is the group mean of re-scaled data to have median equal to 1.
[b]Mean values in green were decreased, whereas in red were increased with *P*- and *q*- values < 0.05. P values were calculated from one way Anova; *q*- values were used to estimate the false discovery rate (FDR) in multiple comparisons.

Table S3. Serum metabolites related to carbohydrate and energy metabolism that were altered in cats undergoing weight loss[a,b]

Metabolic Pathway	Metabolite	Fold change[a, b]					
		wk 1 / wk 0	wk 2 / wk 0	wk 4 / wk 0	wk 8 / wk 0	wk 12 / wk 0	wk 16 / wk 0
Carbohydrates							
Glycolysis, GNG, and Pyruvate Metabolism	lactate	**0.68**	**0.68**	**0.68**	**0.8**	0.84	**0.74**
	glycerate	**0.88**	**0.81**	**0.85**	**0.85**	**0.84**	**0.84**
Pentose Metabolism	ribose	0.68	**0.52**	**0.48**	**0.59**	0.73	**0.59**
	arabitol	0.98	0.96	0.8	**0.81**	0.92	**0.8**
Fructose, Mannose and Galactose Metabolism	fructose	**1.15**	**1.22**	**1.18**	**1.21**	**1.29**	**1.25**
	mannose	**1.27**	**1.23**	**1.19**	**1.21**	**1.18**	**1.11**
Aminosugar Metabolism	glucuronate	1.03	0.98	**0.93**	**0.9**	**0.91**	**0.85**
	N-acetylneuraminate	**0.74**	**0.72**	**0.61**	**0.62**	**0.56**	**0.54**
	erythronate*	**1.12**	**1.11**	1.08	1.07	**1.14**	1.07
Energy							
TCA Cycle	citrate	1.09	1.07	0.99	0.98	1.05	**1.09**
	alpha-ketoglutarate	0.83	**0.81**	0.85	0.94	0.92	0.95
	succinylcarnitine	1.18	1.23	**1.33**	**1.51**	**1.65**	**1.53**
	succinate	**0.81**	**0.83**	0.84	0.91	0.91	**0.83**
	fumarate	**0.56**	**0.54**	**0.59**	**0.75**	**0.7**	**0.69**
	tricarballylate	1.04	0.91	1.6	0.7	**0.54**	**0.48**
Oxidative Phosphorylation	phosphate	**0.93**	**0.93**	**0.91**	**0.92**	**0.9**	**0.88**

[a]For each metabolite, mean value is the group mean of re-scaled data to have median equal to 1.
[b]Mean values in green were decreased, whereas in red were increased with *P*- and *q*- values < 0.05. P values were calculated from one way Anova; *q*- values were used to estimate the false discovery rate (FDR) in multiple comparisons.

Table S4. Serum metabolites related to nucleotide, xenobiotic, and cofactor and vitamin metabolism that were altered in cats undergoing weight loss[a,b]

Metabolic Pathway	Metabolite	Fold change					
		wk 1 / wk 0	wk 2 / wk 0	wk 4 / wk 0	wk 8 / wk 0	wk 12 / wk 0	wk 16 / wk 0
	Nucleotide						
Purine Metabolism, (Hypo)Xanthine/ Inosine containing	inosine	1.02	0.96	1.01	0.97	0.97	**0.89**
	hypoxanthine	0.81	**0.74**	**0.72**	**0.78**	**0.8**	**0.7**
	xanthine	**0.48**	**0.44**	**0.51**	**0.66**	**0.63**	0.7
	2'-deoxyinosine	**0.46**	**0.51**	**0.45**	**0.55**	0.74	**0.63**
	urate	**0.74**	**0.7**	**0.77**	**0.84**	**0.74**	**0.84**
	allantoic acid	1.21	1.25	1.26	**0.81**	1.26	1.05
Purine Metabolism, Adenine containing	N6-methyladenosine	1.1	**1.33**	**1.6**	**1.66**	**1.62**	**1.63**
	N6-carbamoylthreonyladenosine	1.02	1.12	**1.27**	**1.36**	**1.3**	**1.22**
Purine Metabolism, Guanine containing	guanosine	0.98	0.83	0.78	0.9	0.91	**0.76**
	guanine	0.68	**0.58**	**0.49**	**0.65**	**0.62**	**0.61**
	7-methylguanine	**1.08**	1.06	1.07	**1.06**	**1.11**	**1.12**
Pyrimidine Metabolism, Orotate containing	orotate	1.1	1.08	1.17	**1.26**	**1.38**	**1.2**
	orotidine	0.96	1.15	1.29	1.46	**1.5**	1.11
Pyrimidine Metabolism, Uracil containing	uridine	0.82	**0.69**	**0.68**	0.77	**0.7**	**0.61**
	uracil	**0.53**	**0.46**	**0.6**	**0.65**	**0.69**	**0.62**
	pseudouridine	1.08	1.07	1.06	1.05	**1.09**	1.03
	2'-deoxyuridine	**0.63**	**0.6**	**0.68**	0.86	0.79	0.75
	3-ureidopropionate	1.08	1.11	1.2	**1.21**	**1.25**	1.12
	N-acetyl-beta-alanine	0.88	0.91	**0.85**	0.91	0.93	**0.87**
Pyrimidine Metabolism, Cytidine containing	cytidine 5'-monophosphate (5'-CMP)	0.63	0.72	0.39	0.66	**0.52**	**0.4**
	cytidine	0.89	0.86	**0.8**	**0.79**	**0.67**	**0.68**
	5-methylcytidine	0.91	**0.9**	**0.84**	**0.76**	1	**1.16**
	N4-acetylcytidine	1.13	**1.11**	**1.27**	**1.31**	**1.21**	**1.22**
	2'-deoxycytidine	1.02	1.02	1.06	1.05	**1.11**	**1.12**
	5-methyl-2'-deoxycytidine	0.95	0.98	0.99	**0.84**	0.93	1.02
Pyrimidine Metabolism, Thymine containing	3-aminoisobutyrate	1.11	**1.25**	1.13	**1.29**	**1.19**	**1.18**
	Xenobiotics						
Benzoate Metabolism	2-hydroxyhippurate (salicylurate)	0.89	0.93	0.88	**0.73**	**0.64**	**0.62**
	3-hydroxyhippurate	0.95	0.85	0.52	0.5	0.49	**0.43**
	4-hydroxyhippurate	0.87	0.87	**0.58**	**0.58**	**0.58**	**0.5**
	mandelate	1.08	0.99	0.86	0.76	**1.3**	1.27

Table S4 (cont.)

	3-methyl catechol sulfate (1)	0.8	0.94	**0.68**	**0.61**	0.92	1.04
	4-methylcatechol sulfate	0.95	1.13	**0.63**	1	**0.64**	0.82
	4-ethylphenylsulfate	**0.41**	**0.46**	**0.24**	**0.34**	**0.26**	**0.22**
	4-vinylphenol sulfate	**0.49**	**0.36**	**0.31**	**0.24**	**0.19**	**0.19**
	3-(2-hydroxyphenyl)propionate	0.82	0.75	**0.63**	0.58	**0.46**	0.66
	3-methoxycatechol sulfate (2)	**0.45**	0.67	**0.38**	**0.55**	**0.46**	**0.46**
	methyl-4-hydroxybenzoate sulfate	0.35	**0.22**	**0.14**	**0.18**	**0.24**	**0.49**
Food Component/ Plant	2-piperidinone	0.83	0.81	**0.74**	**0.81**	0.85	**0.76**
	gluconate	**0.64**	**0.63**	**0.76**	0.71	**0.37**	**0.34**
	cinnamoylglycine	1.58	1.73	1.09	**2.27**	1.32	**2**
	equol sulfate	0.67	0.84	**0.47**	**0.41**	**0.17**	**0.07**
	ergothioneine	0.85	**0.8**	**0.79**	**0.74**	**0.64**	**0.57**
	ferulic acid 4-sulfate	0.37	0.65	0.35	**0.08**	**0.14**	**0.23**
	indoleacrylate	0.81	**0.85**	**0.84**	**0.7**	**0.72**	**0.73**
	thymol sulfate	**0.72**	**0.57**	**0.41**	**0.22**	**0.14**	**0.1**
	4-allylphenol sulfate	1.05	1.06	1.14	**1.28**	**1.32**	**1.44**
	methyl glucopyranoside (alpha + beta)	0.93	0.92	0.8	**0.63**	**0.55**	**0.39**
	4-vinylguaiacol sulfate	**1.8**	1.34	1.2	1	1	0.81
	pyrraline	**0.82**	**0.83**	**0.72**	**0.6**	**0.72**	**0.78**
	eugenol sulfate	**0.82**	0.94	0.92	0.83	**0.65**	**0.51**
Drug	4-acetylphenol sulfate	0.4	0.44	**0.44**	**0.26**	**0.89**	**0.29**
	6-oxopiperidine-2-carboxylic acid	1.13	1.11	1.04	1.04	1.16	1
	hydroquinone sulfate	0.7	0.78	**0.46**	**0.47**	**0.44**	**0.51**
	salicylate	0.73	0.71	**0.65**	**0.5**	**0.34**	**0.31**
Chemical	1,2-propanediol	0.83	**0.67**	0.83	0.76	0.78	0.78
	2-pyrrolidinone	0.97	0.89	0.94	0.92	1.01	**1.64**
	O-sulfo-L-tyrosine	**0.84**	**0.8**	**0.76**	**0.77**	**0.8**	**0.69**
	ethyl glucuronide	**0.17**	**0.24**	**0.31**	**0.28**	0.91	1.36
	2-aminophenol sulfate	0.83	0.91	**0.67**	**0.63**	**0.54**	**0.6**
	2-ethylhexanoate	1.05	1.05	1.1	**1.1**	1.04	**1.09**
	2-hydroxyisobutyrate	1.18	**1.27**	**1.34**	**1.63**	**1.9**	**1.87**
	dimethyl sulfone	**1.4**	**1.31**	1.29	1.2	0.94	0.94
	ectoine	1.05	1.03	1.14	**1.22**	**1.23**	1.12
	3-hydroxypyridine sulfate	**0.69**	**0.74**	**0.54**	**0.45**	**0.65**	0.78
Vitamins and Cofactors							
Nicotinate and Nicotinamide Metabolism	nicotinamide	**0.43**	**0.44**	**0.61**	**0.62**	**0.57**	**0.55**
	1-methylnicotinamide	0.83	0.7	0.7	0.58	0.7	**0.37**
	trigonelline (N'-methylnicotinate)	0.99	0.92	0.93	**0.83**	**0.81**	0.9
	N1-Methyl-2-pyridone-5-carboxamide	1.06	0.99	0.95	0.89	0.83	**0.59**
Riboflavin Metabolism	riboflavin (Vitamin B2)	0.95	**0.81**	**0.75**	**0.69**	**0.61**	**0.77**

Table S4 (cont.)

Pantothenate and CoA Metabolism	pantothenate	0.98	0.94	0.95	0.96	0.92	**0.86**
Ascorbate and Aldarate Metabolism	threonate	0.98	0.99	0.98	0.96	0.97	**0.91**
	oxalate (ethanedioate)	0.92	0.9	0.97	**0.89**	0.92	**0.88**
	gulonic acid*	1.14	1.07	1.01	0.92	0.95	**0.88**
Tocopherol Metabolism	alpha-tocopherol	0.94	0.97	0.92	**0.89**	0.91	0.94
	delta-tocopherol	0.68	0.69	**0.59**	0.71	**0.55**	**0.63**
	gamma-tocopherol	0.82	0.89	**0.76**	**0.64**	**0.55**	**0.56**
	alpha-CEHC sulfate	1.07	1.06	1.09	**1.44**	**1.44**	**1.53**
Hemoglobin and Porphyrin Metabolism	bilirubin (Z,Z)	1.04	0.74	0.87	**0.58**	0.87	0.71
	bilirubin (E,E)*	2.06	1.58	1.18	0.85	1.63	**1.91**
	biliverdin	1.12	0.86	0.94	**0.42**	0.83	0.66
Vitamin B6 Metabolism	pyridoxine (Vitamin B6)	**0.72**	**0.74**	**0.69**	**0.74**	**0.54**	**0.46**
	pyridoxal	1.39	1.42	**1.55**	1.39	1.12	**1.42**
	pyridoxate	1.19	**1.21**	1.16	1.06	1.01	0.96

[a]For each metabolite, mean value is the group mean of re-scaled data to have median equal to 1.
[b]Mean values in green were decreased, whereas in red were increased with *P*- and *q*- values < 0.05. P values were calculated from one way Anova; *q*- values were used to estimate the false discovery rate (FDR) in multiple comparisons.

Table S5. Correlation coefficients (r) between fasted serum chemistry measures and metabolites[a]

Metabolite	Cr	BUN	TP	GLC	CHOL	TG	ALB	GLOB	ALP	ALT
					**Lipid Metabolism**					
12-hete	0.25	0.49	-0.02	0.16	0.02	-0.08	0.06	-0.04	-0.36	-0.09
Thromboxane B2	0.42	0.50	0.06	0.34	-0.08	-0.16	0.12	0.02	-0.38	-0.07
N-oleoyltaurine	-0.28	0.01	-0.15	0.07	-0.15	0.11	0.38	-0.31	-0.28	-0.11
N-stearoyltaurine	**-0.66**	-0.42	0.31	-0.31	0.17	**0.58**	0.17	0.27	0.15	-0.10
Butyrylcarnitine	**-0.71**	-0.36	0.07	-0.31	-0.07	**0.52**	0.18	0.01	0.20	0.00
Methylmalonate (MMA)	**0.63**	**0.60**	0.12	0.47	-0.14	-0.31	0.17	0.06	-0.31	-0.13
Hydroxybutyrylcarnitine	-0.22	**-0.58**	0.44	-0.34	0.25	0.22	0.07	0.45	0.49	-0.10
2-aminoheptanoate	-0.28	**-0.54**	0.07	0.02	0.12	0.13	0.39	-0.08	0.02	-0.18
3-carboxy-4-methyl-5-propyl-2-furanpropanoate	0.29	**0.75**	0.27	0.12	-0.11	0.09	0.09	0.26	-0.33	0.13
Eicosanodioate	-0.25	-0.49	0.06	0.17	-0.29	0.24	0.43	-0.10	0.08	0.14
Hexadecanedioate	**-0.59**	-0.22	0.13	-0.02	-0.09	**0.51**	0.31	0.02	-0.14	0.05
Octadecanedioate	-0.41	-0.45	0.11	0.11	-0.18	0.39	0.33	-0.01	0.00	0.14
Tetradecanedioate	-0.50	-0.13	0.17	-0.01	0.17	0.42	0.25	0.08	-0.28	-0.05
12,13-dihome	**-0.58**	0.23	0.10	-0.22	-0.19	**0.55**	0.18	0.03	-0.17	0.16
13-hode + 9-hode	**-0.59**	0.16	0.18	-0.40	-0.16	**0.68**	0.03	0.18	-0.07	0.20
16-hydroxypalmitate	**-0.71**	-0.26	0.17	-0.46	-0.30	**0.68**	0.02	0.18	0.47	0.18
2-hydroxydecanoate	**-0.73**	-0.14	0.35	-0.43	0.05	**0.75**	0.09	0.34	0.18	0.13
2-hydroxyoctanoate	**-0.70**	-0.06	0.37	-0.39	0.03	**0.73**	0.18	0.33	0.14	0.11
3-hydroxydecanoate	**-0.59**	-0.27	0.22	-0.45	-0.02	**0.62**	0.12	0.19	0.27	-0.03
3-hydroxylaurate	**-0.71**	-0.20	0.20	-0.42	-0.06	**0.76**	0.06	0.20	0.11	0.09
3-hydroxymyristate	-0.49	-0.17	0.26	-0.31	0.23	**0.54**	0.04	0.27	-0.11	0.05
Glycerol	**-0.54**	**-0.58**	-0.02	-0.20	-0.35	0.48	0.09	-0.06	0.47	0.24
Scyllo-inositol	0.03	0.02	0.44	0.24	0.19	0.26	0.28	0.37	-0.13	0.02
3-hydroxybutyrate (BHBA)	-0.23	-0.47	0.48	-0.39	0.35	0.24	0.03	**0.51**	0.35	-0.13
10-heptadecenoate (17:1n7)	**-0.53**	-0.11	-0.05	-0.29	-0.37	0.42	0.07	-0.09	0.06	0.15
10-nonadecenoate (19:1n9)	-0.39	-0.15	-0.23	-0.16	-0.37	0.18	0.08	-0.28	0.04	0.08
Arachidate (20:0)	-0.33	0.16	-0.18	-0.24	-0.13	0.16	-0.07	-0.17	-0.20	0.09
Cis-vaccenate (18:1n7)	-0.34	-0.02	-0.13	-0.12	-0.33	0.18	0.17	-0.21	-0.10	-0.08
Eicosenoate (20:1n9 or 11)	-0.37	-0.10	-0.17	-0.30	-0.25	0.18	0.00	-0.18	0.02	0.07
Erucate (22:1n9)	-0.15	0.22	-0.28	-0.23	0.03	-0.06	-0.17	-0.24	-0.13	-0.16
Margarate (17:0)	-0.17	0.25	-0.14	-0.08	-0.23	0.11	0.03	-0.16	-0.30	0.13
Myristate (14:0)	**-0.70**	**-0.51**	0.13	-0.44	-0.22	**0.60**	-0.02	0.15	0.47	0.13
Myristoleate (14:1n5)	**-0.66**	-0.45	0.16	-0.40	-0.29	**0.64**	0.05	0.15	0.45	0.20
Oleate (18:1n9)	-0.22	0.04	-0.03	-0.18	-0.33	0.20	0.12	-0.09	-0.10	0.14
Palmitoleate (16:1n7)	**-0.58**	-0.26	0.10	-0.40	-0.37	**0.55**	0.05	0.09	0.29	0.22
1-linoleoylglycerophosphoethanolamine	0.14	0.39	-0.08	0.20	0.02	-0.09	0.08	-0.12	**-0.52**	0.05

Table S5 (cont.)

1-linoleoylglycerophosphoinositol	-0.14	0.18	-0.46	0.05	0.17	-0.21	0.05	**-0.51**	**-0.56**	-0.21
1-oleoylglycerophosphoethanolamine	0.04	0.33	-0.26	0.20	-0.01	-0.12	0.18	-0.35	**-0.57**	-0.03
1-palmitoylplasmenylethanolamine	0.25	0.46	-0.02	0.17	-0.01	-0.10	0.02	-0.03	-0.33	-0.02
2-palmitoleoylglycerophosphocholine	**-0.54**	0.03	-0.14	-0.20	-0.06	0.33	0.14	-0.20	-0.22	0.07
Oleoyl-linoleoyl-glycerophosphoinositol	0.05	**0.63**	-0.17	0.01	0.20	-0.10	0.05	-0.21	**-0.56**	-0.13
Palmitoyl-linoleoyl-glycerophosphocholine	0.03	-0.12	-0.07	0.01	**0.59**	-0.21	-0.08	-0.05	-0.17	-0.38
Palmitoyl-linoleoyl-glycerophosphoinositol	-0.02	**0.54**	-0.16	-0.02	0.22	-0.06	0.02	-0.19	-0.50	-0.14
Stearoyl-arachidonoyl-glycerophosphocholine	-0.41	-0.18	0.36	-0.37	0.32	0.40	-0.01	0.39	0.25	-0.19
Stearoyl-linoleoyl-glycerophosphocholine	0.09	0.44	0.24	-0.09	0.46	0.08	-0.17	0.33	-0.28	-0.06
Stearoyl-linoleoyl-glycerophosphocholine	0.22	0.47	0.08	0.18	0.18	-0.12	-0.15	0.14	-0.27	-0.08
10-undecenoate (11:1n1)	0.20	-0.33	-0.15	0.31	-0.32	-0.24	0.50	-0.36	-0.01	-0.03
5-dodecenoate (12:1n7)	**-0.67**	-0.39	0.03	-0.29	-0.23	**0.56**	0.14	-0.02	0.26	0.07
Caprate (10:0)	**-0.65**	-0.29	0.18	-0.17	-0.13	**0.59**	0.25	0.10	0.11	0.08
Laurate (12:0)	**-0.72**	-0.37	0.13	-0.38	-0.26	**0.66**	0.10	0.10	0.31	0.14
Mevalonate	-0.36	0.22	-0.18	-0.36	0.17	0.07	-0.30	-0.08	-0.26	0.04
1-arachidonylglycerol	-0.36	**-0.54**	-0.24	-0.26	-0.25	0.11	0.05	-0.28	0.43	0.04
1-docosahexaenoylglycerol	-0.38	-0.46	-0.18	-0.30	-0.26	0.23	-0.03	-0.18	0.48	0.14
1-linoleoylglycerol (1-monolinolein)	-0.31	-0.45	-0.37	-0.23	-0.15	0.02	0.01	-0.40	0.24	-0.05
1-oleoylglycerol (1-monoolein)	-0.39	-0.39	-0.35	-0.18	-0.29	0.15	0.17	-0.45	0.22	0.04
1-palmitoylglycerol (1-monopalmitin)	-0.29	**-0.61**	-0.30	-0.22	-0.25	0.01	0.08	-0.36	0.41	0.01
2-docosahexaenoylglcyerol	-0.40	-0.31	-0.29	-0.24	-0.18	0.10	-0.08	-0.29	0.35	0.08
2-linoleoylglycerol (2-monolinolein)	-0.30	-0.37	-0.40	-0.04	-0.24	-0.03	0.08	-0.47	0.25	-0.03
2-oleoylglycerol (2-monoolein)	-0.35	-0.22	-0.23	-0.08	-0.24	0.23	0.23	-0.34	0.07	0.08
Glycerophosphoethanolamine	-0.09	0.17	0.01	0.05	0.23	0.07	0.11	-0.04	-0.41	-0.12
Adrenate (22:4n6)	**-0.56**	-0.20	0.02	-0.30	-0.24	0.43	0.10	-0.02	0.22	0.13
Dihomo-linoleate (20:2n6)	-0.46	-0.06	-0.14	-0.36	-0.31	0.32	-0.01	-0.15	0.12	0.16
Dihomo-linolenate (20:3n3 or n6)	**-0.63**	0.17	-0.21	-0.31	-0.28	0.46	0.07	-0.26	-0.02	0.09
Docosahexaenoate (DHA; 22:6n3)	**-0.73**	-0.42	-0.10	-0.41	-0.05	0.38	0.12	-0.16	0.26	-0.03
Docosapentaenoate (n3 DPA; 22:5n3)	**-0.54**	-0.22	-0.17	-0.40	-0.13	0.26	0.01	-0.19	0.17	0.04
Docosapentaenoate (n6 DPA; 22:5n6)	**-0.64**	-0.48	-0.19	-0.41	-0.17	0.27	0.07	-0.23	0.45	-0.02
Stearidonate (18:4n3)	**-0.64**	-0.11	-0.05	-0.22	-0.25	0.48	0.20	-0.13	-0.07	0.13
Taurolithocholate 3-sulfate	-0.39	-0.16	-0.33	-0.35	-0.01	0.04	0.05	-0.38	0.15	-0.04
Arachidoyl sphingomyelin	0.03	-0.16	0.01	0.15	**-0.53**	0.14	0.09	-0.03	0.40	0.32
Myristoyl sphingomyelin	**-0.53**	**-0.66**	0.00	-0.04	0.12	0.20	0.14	-0.06	0.15	-0.15
Palmitoleoyl sphingomyelin	-0.12	-0.37	0.08	-0.19	**0.54**	-0.11	-0.09	0.12	0.09	-0.22
Beta-sitosterol	0.23	0.27	-0.43	0.18	0.30	-0.37	-0.19	-0.39	**-0.53**	-0.06
Campesterol	0.01	0.16	-0.05	0.15	0.50	-0.09	0.06	-0.08	**-0.57**	-0.25
Cholestanol	-0.30	-0.16	0.24	-0.18	**0.73**	0.26	0.05	0.24	-0.25	-0.07
Cholesterol	-0.20	-0.17	0.31	-0.15	**0.85**	0.12	-0.05	0.36	-0.12	-0.42

Table S5 (cont.)

**Amino Acid Metabolism**										
N-acetylaspartate (NAA)	**0.61**	0.40	0.10	0.19	-0.17	-0.26	0.18	0.04	-0.20	0.15
Creatine	-0.06	-0.20	0.47	0.19	0.41	0.20	-0.04	**0.53**	-0.17	0.00
Creatine phosphate	0.29	0.17	0.40	**0.63**	0.11	0.06	0.21	0.36	-0.30	0.03
Creatinine	**0.95**	0.38	0.09	**0.52**	-0.16	**-0.54**	0.11	0.05	-0.22	0.00
Felinine	0.10	0.00	0.33	0.29	**-0.51**	0.24	0.24	0.27	0.15	0.27
Gamma-glutamylfelinylglycine*	0.23	0.30	0.29	0.38	**-0.52**	0.25	0.31	0.19	-0.16	0.37
N-acetylfelinine*	**0.57**	**0.62**	0.13	0.47	**-0.56**	-0.05	0.41	-0.01	-0.37	0.32
Ophthalmate	0.19	**-0.61**	0.09	0.12	-0.21	-0.15	0.15	0.03	**0.57**	0.09
Betaine	**0.54**	0.38	0.17	0.31	-0.29	-0.15	0.20	0.11	-0.08	-0.02
Serine	**-0.64**	**-0.52**	0.09	**-0.53**	-0.08	0.44	-0.17	0.16	**0.72**	0.06
3-hydroxy-2-ethylpropionate	-0.24	**-0.63**	0.06	-0.20	-0.17	0.20	0.03	0.05	0.50	0.23
3-hydroxyisobutyrate	**-0.52**	-0.42	-0.25	-0.31	0.08	0.12	-0.29	-0.16	0.33	-0.11
3-methyl-2-oxobutyrate	-0.27	-0.42	-0.08	-0.14	-0.02	0.13	0.00	-0.09	0.37	0.14
6-hydroxynorleucine	-0.22	-0.47	0.05	0.06	-0.34	0.19	0.44	-0.12	0.27	-0.03
Allo-isoleucine	-0.22	-0.39	-0.03	-0.27	-0.02	0.09	-0.03	-0.02	**0.53**	-0.14
Alpha-hydroxyisocaproate	-0.10	**-0.64**	-0.02	0.00	0.29	-0.10	-0.12	0.02	0.32	-0.09
Beta-hydroxyisovalerate	0.17	**0.51**	-0.01	-0.02	-0.45	-0.01	0.05	-0.03	0.08	0.21
Ethylmalonate	-0.36	0.11	0.06	-0.19	-0.23	0.33	0.46	-0.12	0.08	-0.05
Isobutyrylcarnitine	-0.43	**-0.52**	0.35	-0.37	-0.06	**0.51**	0.02	0.38	**0.67**	0.11
Isovalerylcarnitine	**-0.64**	-0.43	-0.03	-0.17	-0.11	0.43	0.09	-0.07	0.32	0.04
N-acetylisoleucine	0.31	0.25	0.22	**0.51**	-0.45	0.11	**0.62**	-0.01	-0.21	0.19
Valine	-0.40	-0.08	-0.04	-0.25	-0.25	0.42	0.14	-0.09	0.35	0.18
2-aminoadipate	**0.57**	0.10	0.39	0.19	0.23	-0.25	-0.06	0.45	0.14	-0.03
3-methylglutarylcarnitine (1)	-0.18	**-0.55**	0.04	-0.26	-0.39	0.20	0.05	0.03	**0.77**	0.26
Glutarylcarnitine (C5)	0.05	-0.42	0.01	-0.01	-0.26	-0.01	0.27	-0.10	0.50	0.10
Lysine	0.16	0.16	0.17	-0.09	-0.39	0.17	-0.07	0.21	**0.51**	0.33
N6-acetyllysine	**0.60**	0.35	-0.07	0.17	0.05	-0.43	-0.23	0.02	0.10	-0.08
N-6-trimethyllysine	**0.67**	0.38	0.04	0.48	-0.38	-0.35	0.22	-0.04	-0.04	-0.03
Pipecolate	0.32	**0.56**	0.09	0.14	-0.23	-0.07	0.13	0.04	0.03	-0.07
2-aminobutyrate	-0.22	**-0.54**	-0.17	-0.19	-0.20	0.01	-0.17	-0.12	**0.60**	0.13
Cystathionine	0.04	0.16	0.31	0.07	0.26	0.08	0.20	0.26	-0.43	-0.06
Hypotaurine	-0.06	0.17	**0.59**	0.21	0.18	0.46	0.25	**0.54**	-0.24	0.09
N-acetyltaurine	0.18	0.42	0.04	0.01	0.30	-0.15	-0.06	0.07	-0.26	-0.22
N-formylmethionine	0.45	0.05	-0.47	0.15	-0.05	**-0.57**	-0.11	-0.47	-0.07	0.03
Taurine	-0.03	0.31	0.06	-0.04	0.30	0.01	-0.07	0.09	-0.21	-0.24
3-(3-hydroxyphenyl)propionate	-0.09	0.41	0.40	0.01	-0.01	0.40	0.23	0.35	-0.17	0.06
3-(4-hydroxyphenyl)lactate	0.05	-0.19	-0.44	-0.14	0.10	-0.30	**-0.56**	-0.26	0.32	0.09
3-[3-(sulfooxy)phenyl]propanoic acid	-0.03	0.47	0.33	0.04	-0.06	0.34	0.26	0.26	-0.33	0.09

Table S5 (cont.)

3-methoxytyrosine	-0.40	**-0.58**	0.19	-0.08	-0.12	0.37	0.15	0.15	0.35	-0.04
4-hydroxycinnamate sulfate	0.24	**0.65**	0.01	0.13	-0.10	-0.11	0.05	-0.01	-0.47	0.06
4-hydroxyphenylacetyl glycine	-0.35	-0.30	0.15	-0.39	**0.67**	0.12	-0.09	0.19	-0.01	-0.32
4-hydroxyphenylpyruvate	-0.04	-0.11	-0.21	-0.22	0.42	-0.21	**-0.57**	-0.01	0.30	-0.06
Dopamine sulfate (2)	0.22	0.34	-0.23	0.33	-0.24	-0.23	0.44	-0.42	**-0.51**	-0.03
Gentisate	0.10	**0.59**	0.33	0.22	-0.36	0.29	0.31	0.24	-0.18	0.27
P-cresol sulfate	0.16	0.38	0.22	0.00	**-0.51**	0.14	0.23	0.15	0.24	0.21
Phenyllactate (PLA)	-0.05	0.01	-0.20	-0.26	0.43	-0.13	**-0.55**	0.00	0.03	0.07
Tyrosine	**-0.50**	0.08	-0.23	-0.26	-0.16	0.39	0.04	-0.27	0.08	0.10
N-acetylputrescine	0.04	0.17	0.29	0.15	0.19	0.09	0.47	0.14	-0.39	-0.23
3-indoxyl sulfate	0.31	**0.60**	0.14	0.10	-0.16	-0.14	0.10	0.11	-0.12	0.14
Anthranilate	0.21	**0.53**	0.23	0.02	-0.25	0.06	0.05	0.23	-0.12	0.14
Indole-3-carboxylic acid	0.37	**0.59**	0.19	0.08	-0.26	-0.03	0.11	0.16	-0.22	0.21
Indoleacetate	**0.53**	**0.67**	0.21	0.25	-0.25	-0.14	0.15	0.17	-0.31	0.18
Indolelactate	0.05	0.08	-0.45	-0.18	0.22	-0.29	**-0.55**	-0.28	0.07	0.08
Indolepropionate	0.31	**0.57**	0.23	-0.01	-0.26	0.03	0.08	0.22	-0.15	0.25
Kynurenine	-0.26	**-0.53**	0.13	-0.22	-0.01	0.17	-0.18	0.21	**0.71**	0.01
N-acetylkynurenine (2)	0.17	0.34	-0.23	0.08	0.31	-0.30	0.10	-0.29	-0.46	-0.22
N-acetyltryptophan	0.20	**0.55**	-0.13	0.10	0.14	-0.15	0.12	-0.18	**-0.57**	0.05
Picolinate	0.41	**0.59**	0.15	0.11	-0.26	-0.07	0.08	0.13	-0.26	0.20
Serotonin (5HT)	0.24	0.07	-0.42	0.29	0.04	-0.39	-0.04	-0.44	-0.39	0.04
Tryptophan	**-0.54**	-0.46	-0.10	-0.35	0.36	0.24	0.14	-0.16	0.13	-0.17
Homocitrulline	-0.20	-0.04	**-0.51**	0.04	**-0.63**	0.04	0.03	**-0.57**	0.24	0.17
Pro-hydroxy-pro	**0.63**	0.09	-0.07	**0.53**	0.07	-0.44	0.11	-0.11	-0.20	-0.22
Urea	0.26	**0.75**	0.04	0.06	-0.05	-0.05	0.05	0.02	-0.37	0.17
**Peptide Metabolism**										
Felinylglycine	0.29	0.35	-0.19	0.40	**-0.70**	-0.05	0.24	-0.30	-0.14	0.29
Prolylglycine	**0.75**	0.28	-0.01	0.44	0.12	**-0.55**	0.10	-0.05	-0.33	-0.09
Valylglycine	0.41	**0.52**	-0.16	0.09	-0.05	-0.28	-0.02	-0.16	-0.21	-0.10
Anserine	**0.60**	0.00	0.34	0.37	0.15	-0.28	-0.02	0.38	-0.05	-0.01
Gamma-glutamyl-2-aminobutyrate	-0.04	**-0.65**	-0.07	-0.16	-0.14	-0.10	-0.08	-0.05	**0.78**	0.01
Gamma-glutamylglutamate	0.23	-0.04	**0.54**	0.20	0.25	0.12	0.14	**0.53**	0.14	-0.08
Gamma-glutamylthreonine	-0.20	0.10	-0.28	-0.09	-0.39	0.14	0.25	-0.40	0.24	-0.07
Gamma-glutamyltryptophan	**-0.52**	-0.47	0.15	-0.41	**0.53**	0.33	-0.10	0.20	0.26	-0.21
**Carbohydrate Metabolism**										
Glucose	**0.52**	0.18	0.13	**0.75**	-0.19	-0.14	0.18	0.08	-0.34	0.21
Arabitol	0.19	**0.51**	-0.10	-0.05	-0.11	-0.11	-0.02	-0.10	0.02	0.06
Ribitol	-0.12	-0.32	0.14	-0.30	**0.79**	-0.05	-0.20	0.23	0.03	-0.37
Ribonate	0.17	-0.10	-0.09	-0.09	**0.66**	-0.29	-0.36	0.05	-0.16	-0.16

Table S5 (cont.)

**Energy Metabolism**										
Alpha-ketoglutarate	**0.51**	0.26	0.08	**0.55**	0.00	-0.18	0.05	0.07	-0.37	0.25
Citrate	0.14	0.23	-0.34	0.05	-0.47	-0.03	0.15	-0.43	0.06	0.17
Succinate	0.40	0.30	0.29	0.44	0.04	0.00	0.22	0.23	-0.25	0.08
**Xenobiotic Metabolism**										
2-hydroxyhippurate (salicylurate)	0.27	**0.67**	-0.15	0.15	-0.36	-0.14	-0.03	-0.15	-0.26	0.25
3-hydroxyhippurate	**-0.61**	-0.23	0.01	-0.23	0.31	0.33	-0.04	0.03	-0.10	-0.12
3-methoxycatechol sulfate (2)	0.16	**0.52**	0.28	0.07	-0.20	0.14	0.12	0.25	-0.22	0.14
3-methyl catechol sulfate (1)	0.42	**0.54**	0.17	0.04	-0.35	-0.06	0.19	0.10	-0.14	0.24
4-hydroxyhippurate	-0.28	0.05	0.47	-0.28	**0.52**	0.43	0.06	0.48	-0.17	-0.08
4-methylcatechol sulfate	0.18	0.48	0.20	0.11	**-0.55**	0.20	0.29	0.10	-0.03	0.31
4-vinylphenol sulfate	-0.02	**0.61**	0.11	-0.02	-0.09	0.17	0.06	0.10	-0.39	0.17
Catechol sulfate	0.01	0.18	0.17	-0.04	**-0.54**	0.25	0.17	0.11	0.21	0.40
Hippurate	0.12	0.35	-0.02	-0.01	**-0.50**	0.08	0.13	-0.08	0.13	0.32
1,2,3-benzenetriol sulfate (2)	-0.14	0.15	0.14	-0.16	**-0.50**	0.34	0.04	0.14	0.32	0.27
2-aminophenol sulfate	0.13	**0.79**	0.15	0.06	-0.18	0.12	0.19	0.09	-0.35	0.12
2-hydroxyisobutyrate	0.18	**-0.52**	0.09	-0.01	-0.33	-0.06	0.02	0.09	**0.63**	0.24
3-hydroxypyridine sulfate	0.28	**0.61**	0.19	0.03	0.01	-0.02	-0.03	0.22	-0.33	0.07
Dimethyl sulfone	0.13	0.49	0.17	0.11	**-0.55**	0.20	0.29	0.07	0.01	0.24
Ectoine	-0.15	**-0.51**	0.11	-0.06	0.15	0.01	0.03	0.11	0.21	-0.12
N-methylpipecolate	0.43	**0.65**	-0.03	0.19	-0.44	-0.13	0.09	-0.07	-0.21	0.32
Sulfate*	-0.44	-0.25	0.25	-0.19	-0.30	**0.57**	**0.25**	**0.18**	0.31	0.22
Hydroquinone sulfate	-0.11	**0.56**	0.25	-0.16	-0.02	0.33	0.11	0.22	-0.23	0.12
Salicylate	0.33	**0.64**	0.22	0.11	-0.24	-0.01	0.13	0.19	-0.29	0.18
2,3-dihydroxyisovalerate	-0.12	-0.02	-0.08	0.03	-0.36	0.27	0.24	-0.18	0.13	0.12
2-piperidinone	0.03	0.12	0.17	0.01	**0.51**	-0.04	-0.08	0.22	-0.20	-0.11
4-allylphenol sulfate	**0.52**	0.28	-0.18	0.22	-0.38	-0.40	-0.18	-0.13	0.05	0.01
4-vinylguaiacol sulfate	0.29	**0.50**	-0.12	0.07	-0.07	-0.26	-0.17	-0.07	-0.22	0.13
Erythritol	-0.49	-0.21	0.15	-0.28	-0.20	**0.51**	**0.16**	**0.11**	0.41	0.08
Eugenol sulfate	**0.51**	**0.73**	0.05	0.28	-0.28	-0.22	0.09	0.01	-0.35	0.18
Gluconate	0.10	0.17	-0.30	-0.11	0.27	-0.24	-0.43	-0.16	-0.15	0.09
Homostachydrine*	**0.65**	0.42	0.07	0.39	-0.16	-0.28	0.16	0.01	-0.16	-0.04
Indoleacrylate	0.33	**0.57**	0.17	-0.02	-0.29	0.01	0.08	0.15	-0.06	0.28
Methyl glucopyranoside (alpha + beta)	-0.03	**0.61**	0.03	-0.11	0.15	0.06	-0.03	0.05	-0.42	-0.07
Pyrraline	0.20	**0.64**	0.20	0.06	0.14	0.00	-0.06	0.24	-0.47	0.04
Stachydrine	**0.66**	**0.52**	**0.08**	**0.51**	0.07	-0.35	0.22	0.00	-0.46	-0.16
**Nucleotide Metabolism**										
Allantoic acid	0.45	**0.50**	-0.15	0.19	-0.24	-0.30	0.11	-0.21	-0.18	-0.08
Allantoin	0.28	**0.60**	0.11	0.09	0.21	-0.08	0.06	0.09	-0.40	-0.16

Table S5 (cont.)

Adenosine	0.29	0.25	-0.19	0.04	0.11	-0.17	-0.19	-0.13	-0.21	0.21
N6-methyladenosine	-0.14	**-0.54**	-0.11	-0.17	0.07	-0.03	-0.13	-0.08	**0.50**	-0.29
7-methylguanine	**0.60**	0.31	0.08	0.23	-0.22	-0.34	0.14	0.03	0.03	-0.02
Guanine	**-0.51**	-0.08	0.00	-0.38	0.00	0.44	0.07	-0.03	0.25	-0.07
5-methyl-2'-deoxycytidine	0.47	0.37	-0.28	0.42	-0.08	-0.41	0.05	-0.32	**-0.54**	0.14
Orotate	-0.08	-0.25	-0.32	-0.23	-0.11	-0.18	-0.20	-0.27	**0.62**	-0.15
Thymine	-0.20	-0.28	**0.51**	**-0.16**	**0.15**	**0.39**	**0.03**	**0.55**	0.26	0.10
**Vitamins and Cofactors**										
Alpha-tocopherol	0.33	0.30	0.18	0.13	**0.60**	-0.18	-0.06	0.22	-0.35	-0.18
Gulonic acid	0.46	**0.75**	0.10	0.13	-0.27	-0.05	0.25	0.01	-0.30	0.11
Oxalate (ethanedioate)	**-0.61**	-0.32	-0.30	-0.39	-0.22	0.25	-0.08	-0.29	0.35	0.20
Pyridoxate	0.49	**0.72**	0.06	0.20	0.01	-0.33	0.12	0.02	-0.27	-0.21
Pyridoxine (Vitamin B6)	**-0.60**	-0.10	0.06	-0.36	0.34	0.38	-0.20	0.14	0.00	-0.11
Threonate	**-0.56**	-0.33	**-0.52**	-0.20	-0.24	0.12	0.02	**-0.57**	0.17	0.02
Trigonelline (N'-methylnicotinate)	-0.17	0.13	-0.05	0.26	0.17	0.03	0.17	-0.12	**-0.52**	-0.10

[a]Bold correlation coefficients (r) with $p<0.0001$ and with strong correlation ($r>0.5$). Cr: creatinine; BUN: blood urea nitrogen; TP: total protein; GLC: glucose; CHOL: cholesterol; TG: triglycerides; ALB: albumin; GLOB: globulin; ALP: alkaline phosphatase; ALT: alanine aminotransferase.

Table S6. Correlation coefficients (r) between DEXA scan results and metabolites[a]

**Metabolite**	FtMass	LnMass	BW	%Fat
		**Lipid Metabolism**		
N-stearoyltaurine	**0.84**	-0.18	**0.77**	**0.87**
butyrylcarnitine	**0.74**	-0.12	**0.69**	**0.79**
hydroxybutyrylcarnitine*	0.47	-0.32	0.38	**0.54**
octanoylcarnitine	**0.59**	-0.23	**0.52**	**0.64**
2-aminooctanoate	**0.63**	-0.13	**0.58**	**0.65**
15-methylpalmitate (isobar with 2-methylpalmitate)	**0.71**	0.02	**0.69**	**0.71**
17-methylstearate	0.49	-0.19	0.43	**0.51**
13-HODE + 9-HODE	**0.61**	0.22	**0.64**	**0.52**
16-hydroxypalmitate	**0.85**	-0.03	**0.81**	**0.81**
2-hydroxydecanoate	**0.91**	0.10	**0.91**	**0.86**
2-hydroxyoctanoate	**0.83**	0.08	**0.82**	**0.81**
3-hydroxydecanoate	**0.73**	-0.01	**0.70**	**0.70**
3-hydroxylaurate	**0.74**	-0.03	**0.71**	**0.72**
alpha-hydroxycaproate	0.46	-0.33	0.38	**0.58**
glycerol	**0.65**	-0.07	**0.62**	**0.66**
3-hydroxybutyrate (BHBA)	0.50	-0.22	0.44	**0.54**
acetoacetate	0.46	-0.32	0.38	**0.54**
10-heptadecenoate (17:1n7)	**0.57**	-0.04	**0.55**	**0.56**
myristate (14:0)	**0.81**	-0.18	**0.75**	**0.81**
myristoleate (14:1n5)	**0.87**	0.00	**0.84**	**0.82**
palmitoleate (16:1n7)	**0.66**	-0.05	**0.63**	**0.63**
stearoyl-arachidonoyl-glycerophosphocholine (2)*	**0.60**	-0.11	**0.56**	**0.61**
stearoyl-linoleoyl-glycerophosphocholine (2)*	-0.49	0.11	-0.45	**-0.51**
5-dodecenoate (12:1n7)	**0.77**	-0.11	**0.72**	**0.76**
caprate (10:0)	**0.68**	-0.04	**0.65**	**0.65**
laurate (12:0)	**0.87**	-0.05	**0.84**	**0.85**
1-arachidonylglycerol	0.49	-0.17	0.44	**0.51**
1-docosahexaenoylglycerol	**0.52**	-0.07	0.49	0.50
adrenate (22:4n6)	**0.66**	-0.01	**0.64**	**0.64**
dihomo-linolenate (20:3n3 or n6)	**0.54**	0.11	**0.55**	**0.54**
docosahexaenoate (DHA; 22:6n3)	**0.73**	-0.18	**0.67**	**0.76**
docosapentaenoate (n3 DPA; 22:5n3)	**0.60**	-0.13	**0.55**	**0.63**
docosapentaenoate (n6 DPA; 22:5n6)	**0.73**	-0.15	**0.67**	**0.75**
stearidonate (18:4n3)	**0.66**	0.06	**0.65**	**0.63**
myristoyl sphingomyelin*	**0.56**	-0.34	0.47	**0.66**
5alpha-pregnan-3beta,20beta-diol monosulfate (1)	-0.24	**0.55**	-0.12	-0.32
beta-sitosterol	**-0.57**	0.13	**-0.52**	**-0.55**
		**Amino Acid Metabolism**		
N-acetylaspartate	**-0.58**	0.10	**-0.54**	**-0.64**
Creatinine	**-0.68**	0.27	**-0.60**	**-0.75**
Gamma-glutamylfelinylglycine	0.06	**0.58**	0.18	-0.11
N-acetylfelinine*	-0.26	**0.62**	-0.13	-0.45
Glutathione, oxidized	0.16	0.28	0.22	0.12
Serine	**0.69**	-0.23	**0.62**	**0.73**
Imidazole lactate	0.48	-0.24	0.41	**0.54**
2-methylbutyrylcarnitine	**0.59**	-0.07	**0.55**	**0.61**
3-hydroxyisobutyrate	0.17	**-0.53**	0.05	0.30
3-methylglutaconate	-0.47	0.38	-0.38	**-0.57**
Ethylmalonate	**0.66**	0.00	**0.64**	**0.66**
Isobutyrylcarnitine	**0.69**	-0.16	**0.64**	**0.70**
Isovalerylcarnitine	**0.67**	-0.13	**0.62**	**0.70**
N-acetylisoleucine	-0.09	**0.53**	0.02	-0.23

Table S6 (cont.)

2-aminoadipate	-0.50	0.07	-0.47	**-0.52**
3-methylglutarylcarnitine	**0.56**	0.02	**0.54**	**0.52**
N6-acetyllysine	**-0.64**	0.05	**-0.61**	**-0.66**
3-methoxytyrosine	0.50	-0.35	0.41	**0.54**
Indoleacetate	**-0.52**	0.35	-0.43	**-0.60**
Picolinate	-0.46	0.26	-0.39	**-0.54**
Tryptophan	0.49	-0.23	0.43	**0.56**
Pro-hydroxy-pro	**-0.58**	0.11	**-0.53**	**-0.60**
Proline	**-0.56**	0.17	**-0.51**	**-0.58**
Trans-4-hydroxyproline	**-0.57**	0.10	**-0.53**	**-0.56**
		**Peptide Metabolism**		
Prolylglycine	**-0.63**	0.13	**-0.58**	**-0.66**
Gamma-glutamyltryptophan	**0.51**	-0.36	0.42	**0.60**
		**Energy Metabolism**		
Alpha-ketoglutarate	-0.49	**0.55**	-0.35	**-0.59**
		**Xenobiotic Metabolism**		
3-(2-hydroxyphenyl)propionate	0.21	0.19	**-0.61**	**0.61**
3-ethylphenylsulfate	-0.10	-0.07	**0.70**	**-0.70**
4-ethylphenylsulfate	0.31	0.32	0.47	-0.46
4-vinylphenol sulfate	**-0.61**	**-0.62**	-0.09	0.09
1,2-propanediol	**0.59**	**0.58**	-0.23	0.24
2-aminophenol sulfate	**-0.73**	**-0.73**	0.11	-0.12
2-ethylhexanoate	-0.10	-0.12	**-0.52**	**0.52**
4-hydroxychlorothalonil	**0.52**	**0.51**	-0.40	0.40
Dimethyl sulfone	**-0.59**	**-0.58**	0.42	-0.42
O-sulfo-L-tyrosine	0.11	0.13	**0.61**	**-0.61**
2-piperidinone	-0.01	-0.03	**-0.67**	**0.66**
Cinnamoylglycine	0.19	0.21	**0.50**	**-0.50**
Ergothioneine	-0.01	0.00	**0.57**	**-0.56**
Erythritol	**0.56**	**0.54**	**-0.62**	**0.62**
Eugenol sulfate	0.39	0.39	-0.06	0.06
Ferulic acid 4-sulfate	0.27	0.28	-0.17	0.16
Gluconate	**0.59**	**0.56**	**-0.72**	**0.73**
Indolin-2-one	**0.59**	**0.60**	-0.01	0.01
N-glycolylneuraminate	**0.52**	**0.51**	-0.41	0.42
Pyrraline	**0.62**	**0.58**	**-0.79**	**0.80**
Stachydrine	**0.58**	**0.60**	0.32	-0.32
Vanillin	0.05	0.10	**0.63**	**-0.64**
		**Nucleotide Metabolism**		
2'-deoxyinosine	**0.55**	-0.20	0.49	**0.58**
7-methylguanine	-0.44	0.02	-0.43	-0.50
N2,N2-dimethylguanine	-0.31	-0.20	-0.34	-0.27
2'-deoxycytidine	0.21	**-0.51**	0.09	0.33
5-methyl-2'-deoxycytidine	**-0.61**	0.44	-0.50	**-0.70**
5-methylcytidine	-0.42	-0.19	-0.45	-0.39
N-acetyl-beta-alanine	**0.51**	-0.09	0.48	**0.52**

[a] Bold correlation coefficients (r) with p<0.05 and with strong correlation (r>0.5). FtMass: total fat mass; LnMass: total lean mass; BW: body weight; % fat: % fat mass.
